# Kiwifruit R2R3-MYB transcription factors and contribution of the novel *AcMYB75* to red kiwifruit anthocyanin biosynthesis

**DOI:** 10.1038/s41598-017-16905-1

**Published:** 2017-12-04

**Authors:** Wenbin Li, Zehong Ding, Mengbin Ruan, Xiaoling Yu, Ming Peng, Yifei Liu

**Affiliations:** 1 Key Laboratory of Biology and Genetic Resources of Tropical Crops, Institute of Tropical Bioscience and Biotechnology, Chinese Academy of Tropical Agricultural Sciences, Haikou, Hainan China; 2 Key Laboratory of Plant Resources Conservation and Sustainable Utilization, South China Botanical Garden, Chinese Academy of Sciences, Guangzhou, Guangdong, China

## Abstract

Red kiwifruit (*Actinidia chinensis*) is a popular fresh fruit with a high market value due to its unique color, caused by anthocyanin accumulation. The R2R3-MYB transcription factors (TFs) have important roles in plant development and anthocyanin metabolism. In this first comprehensive study of R2R3-MYBs in kiwifruit, a total of 93 R2R3-*MYB* genes, including five novel previously unannotated *AcMYB*s, were identified. Their phylogenic relationship, exon-intron structures, and conserved motifs were analyzed. Based on transcriptome data, 60 *AcMYB*s were expressed (FPKM > 1) across seven developmental stages of kiwifruit, revealing five expression patterns. One of the 5 newly identified R2R3 TFs, *AcMYB75*, showed an anthocyanin accumulation-linked expression pattern during fruit development. *AcMYB75* localized to the nucleus and has an active transactivation domain, verifying it as a transcription factor. AcMYB75 protein specifically bound the promoter of the anthocyanin biosynthesis gene *ANS* in yeast one-hybrid system and *in vivo*. In 35 *S:AcMYB75 Arabidopsis* plants, anthocyanin significantly accumulated in leaves, and the expression of anthocyanin biosynthetic genes was greatly up-regulated. Together, these results suggest that *AcMYB75* is involved in anthocyanin biosynthesis in kiwifruit. These findings will increase our understanding of AcMYBs involved in anthocyanin biosynthesis, and also benefit further functional characterization of R2R3-*MYB* genes in kiwifruit.

## Introduction

The rise of colorful flowers and fruits is a crux of plant biology and evolution. Fruit that is more attractive to insects and animals aids in the dissemination of genes and improves the survival rate of progeny^1^. Flower and fruit pigmentation is largely derived from anthocyanins. Anthocyanins are potent antioxidants with health promoting properties, the benefits of which are increasingly of interest to consumers and make colorful fruits more favorable in the market^2^. Plant breeders have picked up nature’s work to augment fruit for the human consumer. Research has led to the understanding that fruit development and anthocyanin biosynthesis are largely controlled by specific transcription factors (TFs)^3,4^, especially R2R3-MYB TFs^5–7^.

The structural characteristics of R2R3-MYB TFs have been extensively described for many species^8–10^. MYB TFs are usually classified as 1R-MYB, R2R3-MYB, 3R-MYB or 4R-MYB, according to the number of conserved DNA-binding domains consisting of imperfect repeats (R) at the N terminus. Each repeat forms the hydrophobic core in a helix-turn-helix (HTH) structure, and the recognition helices in two repeats bind cooperatively to a specific DNA sequence motif. R2R3-MYBs usually possess an N-terminal DNA-binding domain (highly conserved MYB domain; -W-(X_19_)-W-(X_19_)-W-…-F/I-(X_18_)-W-(X_18_)-W-) and a C-terminal transactivation or repression domain^8^.

R2R3-MYB TFs are widely involved in plant development, secondary metabolism, hormone signal transduction, cell apoptosis, disease and abiotic stress responses. Specific R2R3-MYB TFs have regulatory roles during fruit development and ripening and in determining fruit ascorbic acid level, carotenoid, flavonoid, lignin, and terpenoid levels^11–16^. Recent reports on the regulation of anthocyanin accumulation in fruits by R2R3-MYBs include the study of *MdMYB10* in apple^17–20^, *VvMYBA1* and *VvMYBA2* in grape^21,22^, *PavMYB10*.*1* in cherry^23^, *PcMYB10* and *PyMYB10* in pear^24,25^, *FaMYB10* in strawberry^26^ and *AdMYB110a* in kiwifruit flower pigmentation^27^. These studies reveal potential approaches for breeding and/or biotechnological development of diverse colors in horticultural fruits.

Searches of high-throughput genome sequences in different species has identified large numbers of MYB TFs based on conservation of the highly conserved DNA-binding MYB domain^7,28–30^. However, no detailed bioinformatics analysis of the MYB family in kiwifruit (*Actinidia* Lindl.) has been reported. Over the past 30 years, kiwifruit has become a globally important horticultural cash crop. Furthermore, there are niche markets selling uniquely colored kiwifruit at premium prices. Both breeders and molecular biologists are interested in the mechanisms behind such diverse colors, such as yellow and red kiwifruit. The draft kiwifruit genome sequence (http://bioinfo.bti.cornell.edu/cgi-bin/kiwi/home.cgi)^31^ and our high-quality transcriptome data from the red-fleshed Hongyang kiwifruit^32^ provide an excellent opportunity for genome-wide analysis of kiwifruit genes.

In this study, a comprehensive genome-wide analysis was conducted to identify all R2R3-AcMYB TFs in kiwifruit. Analyses included phylogenetic relationship, gene structures, motif features and expression of R2R3 *AcMYBs* during kiwifruit development. Five previously unannotated R2R3 *AcMYB* TFs have been identified. Of these, we were most interested in *AcMYB75*, which showed high sequence similarity to anthocyanin-related R2R3-MYBs from other species and an expression pattern related to anthocyanin accumulation. *AcMYB75* was found to localize to the nucleus and transactivate the anthocyanin synthase (*ANS*) gene promoter in yeast one-hybrid assay, and alter the anthocyanin accumulation when over-expressed in *Arabidopsis*. These results suggest that *AcMYB75* is involved in anthocyanin biosynthesis in kiwifruit.

## Results

### Identification and phylogenetic analysis of R2R3-MYB TFs in kiwifruit

A total of 93 putative R2R3-type MYB transcription factors were identified from the kiwifruit genome sequence (http://bioinfo.bti.cornell.edu/cgi-bin/kiwi/home.cgi) and our transcriptome data^32^ based on the classification in *Arabidopsis thaliana* ^33^. Subsequently, domain detection confirmed that all of the identified R2R3-MYB TFs harbored the conserved MYB domain, which is the basic characteristic of the MYB family. Of these, five R2R3-MYB genes (*AcMYB20*, *AcMYB70*, *AcMYB73*, *AcMYB77*, and *AcMYB75*) were identified through a Tophat-Cufflinks pipeline using our previous transcriptome data and they were unannotated in the public genome database. Subsequently, manual checking with Integrative Genomics Viewer (IGV) further confirmed that the pair-end reads of each of these five genes were mapped on a genome region without gene annotation, respectively, and they were identified in different samples of transcriptome data (Supplementary Fig. S1), strongly supporting that these genes were novel MYBs and not previously identified. The 93 predicted kiwifruit R2R3 MYB proteins varied in length from 177 (AcMYB44) to 1944 (AcMYB76) amino acid residues, ranged in molecular mass from 19.86 to 213.07 kDa, and ranged in isoelectric points from 4.9–9.6 (see Supplementary Table S1).

**Figure 1:**
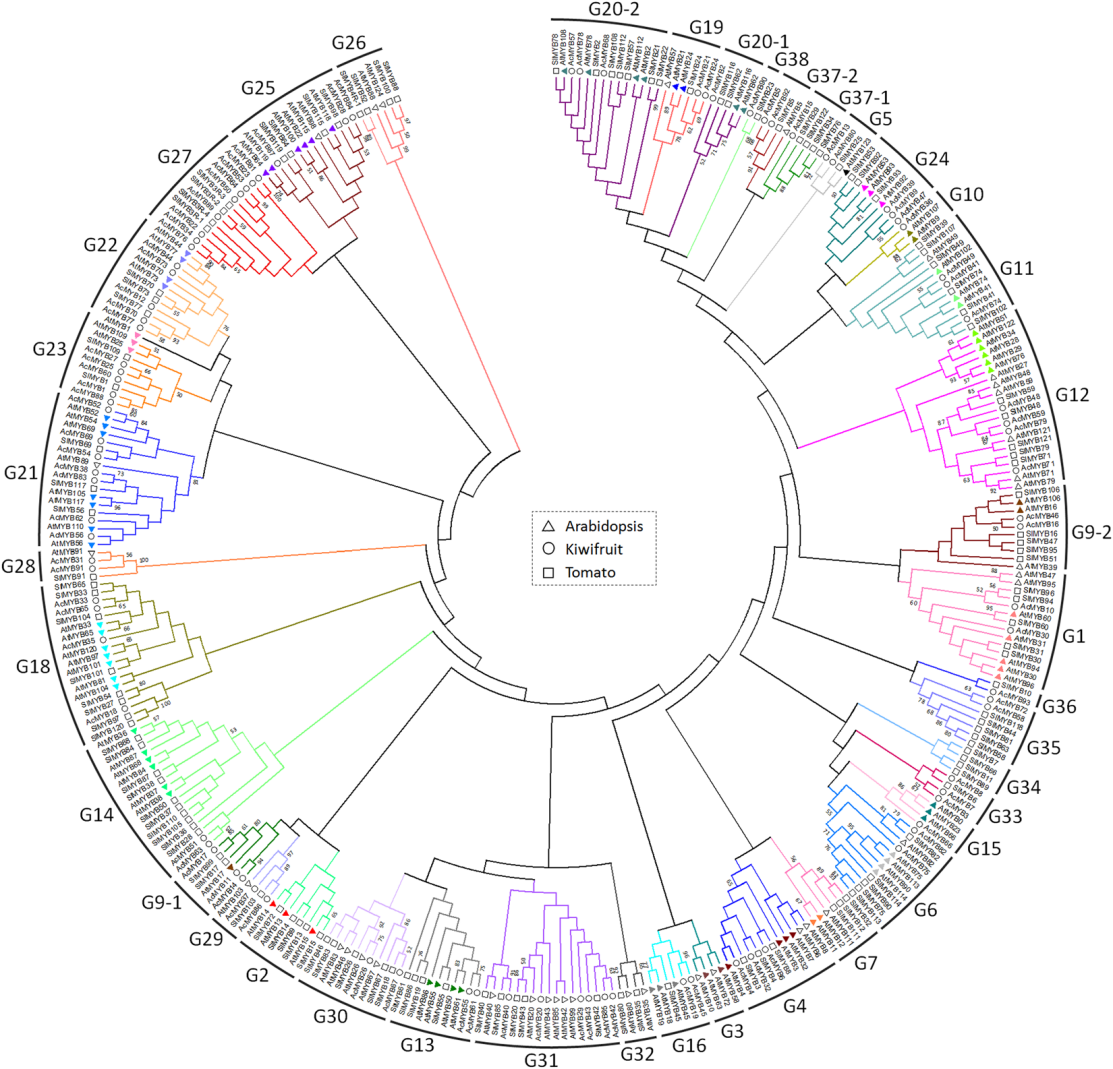
Phylogenetic analysis of R2R3-MYB proteins from kiwifruit, *Arabidopsis*, and tomato. A total of 93 R2R3-MYBs from kiwifruit, 125 R2R3-MYBs from *Arabidopsis* and 127 R2R3-MYBs from tomato were used to create the NJ tree with 1000 bootstraps. The branches with different colors represent different subfamilies of MYB genes. The triangles with different colors represent different groups of *AtMYB* genes according to Stracke *et al*. (2001). The kiwifruit R2R3-MYB proteins are grouped into 36 subgroups (G1 to G38). Of which, the subfamilies from G1 to G25 were designated as previously reported, while the subfamilies from G26 to G38 were designated in our study.

All 93 R2R3 AcMYBs were assigned into 36 subfamilies (G1-G38, except G8 and G17), together with other R2R3-MYB proteins from *Arabidopsis* and tomato (Fig. 1). The members of most known R2R3-MYBs subfamilies from *Arabidopsis* (represented by triangles with different colors) were well separated into different groups, except that the members of the original subfamily G9 and G20 were divided into two groups (G9–1, G9-2, G20-1 and G20-2). The results showed that subfamilies G12, G18, G21, G22, G23 and G24 contained the most AcMYBs (≥4), with the maximum (7) in subfamily G21. There were no AcMYBs in subfamily G2, G3, G7, G26, G32 and G34, indicating an unexpected event during evolution. In G33, G35, G36, G37-1 and G38 families, there were only MYB genes from kiwifruit and tomato, indicating that these MYBs might participate in certain pathways particularly conserved in these two species.

The five newly annotated MYBs in kiwifruit seemed to have close homologs in tomato and/or *Arabidopsis*: *AcMYB20* closely clustered with its *Arabidopsis* homolog *AtMYB20* and its tomato homolog *SlMYB20* in subfamily G31; *AcMYB70*, *AcMYB73*, and *AcMYB77* closely clustered with their homologs in both *Arabidopsis* (*AtMYB77*, *AtMYB44*, *AtMYB73*, *AtMYB70*) and tomato (*SlMYB70*, *SlMYB73*, and *SlMYB77*) in subfamily G22; and AcMYB75 in G6 closely clustered with two functionally characterized genes, *AtMYB75* (*PAP1*) and *AtMYB90* (*PAP2*), which are involved in flavonoid and anthocyanin biosynthesis in *Arabidopsis* ^34^. This similarity suggested that *AcMYB75* might have similar functions in flavonoid and anthocyanin biosynthesis in kiwifruit.

### Gene structure and conserved motifs of kiwifruit MYBs

**Figure 2:**
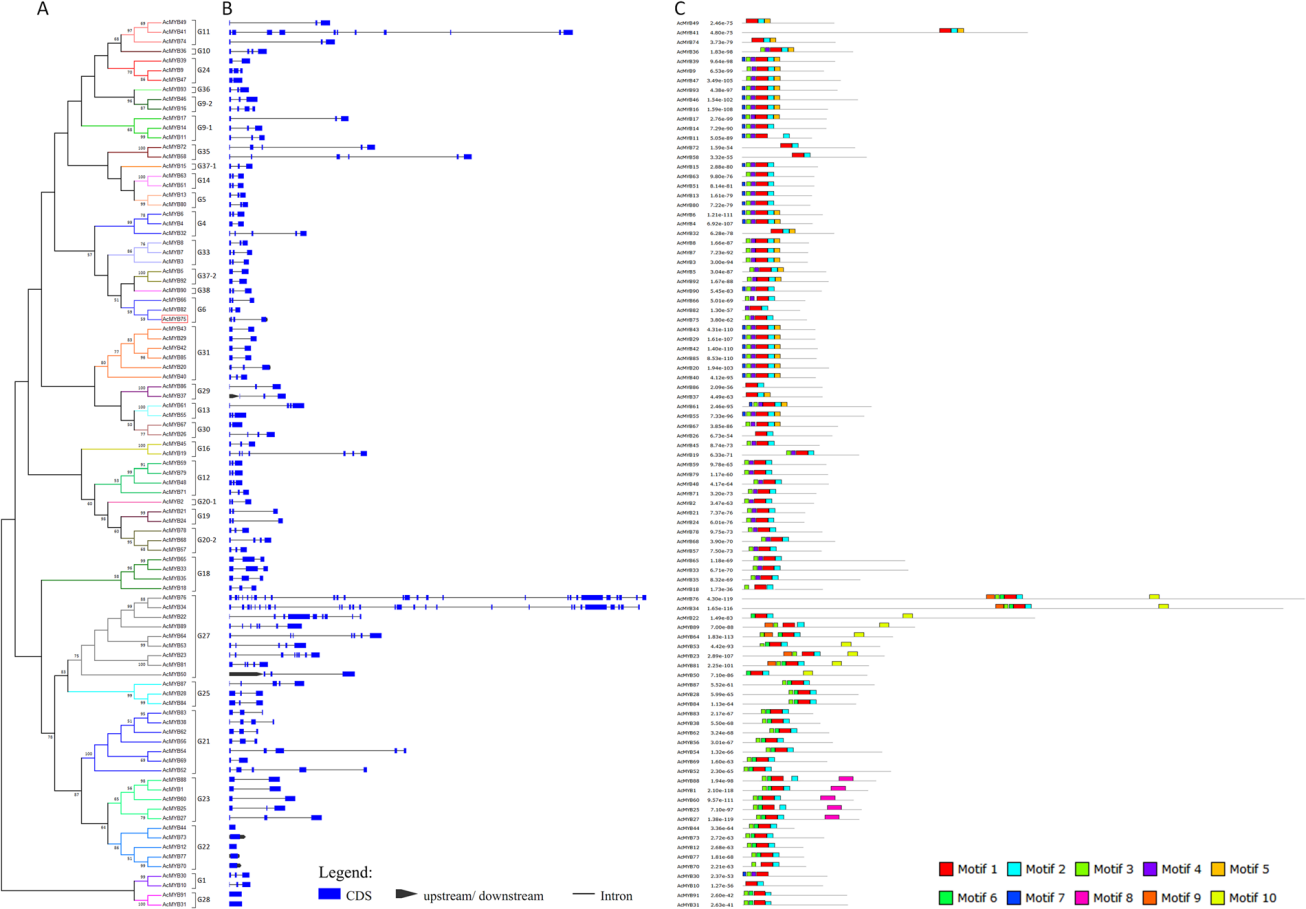
The exon-intron structure of the genes and conserved protein motifs of kiwifruit R2R3-MYB transcription factors. The NJ evolutionary tree (**A**) was created with 1000 bootstraps based on the full-length sequences of the R2R3-AcMYBs. Exon-intron structure (**B**) analyses of R2R3-AcMYB genes were carried out with GSDS, and conserved protein motifs (**C**) were identified by MEME. Lengths of exons, introns and motifs were exhibited proportionally. Grey lines represent the nonconserved sequences, and each motif is indicated by a colored box numbered at the bottom.

The intron/exon structures and protein motifs of the *AcMYB* genes were identified based on their evolutionary relationships to annotated genes (Fig. 2). Gene structure analysis showed that the number of introns in the *AcMYB* genes varied from 0 to 28 (Fig. 2B). Most of subfamilies (18 of 36), i.e. G1, G5, G6-1, G12, G14, G18, and so on, contained 1–2 introns, 10 subfamilies (G10, G35, G4, G9, G13, G16, G20, G25, G21 and G30 contained 2–6 introns, and subfamilies G11 contained 2–10 introns. A few exceptional cases were observed, for example, genes in subfamily G27 had variable numbers of introns, ranging from 2 to 28, while no intron was detected in genes of subfamily G22 and G28. For the five novel identified *AcMYB*s, *AcMYB75* and *AcMYB20* contained three exons and two introns, while *AcMYB70*, *AcMYB73*, and *AcMYB77* contained no intron. AcMYB75 was clustered with AtMYB75 and AtMYB90 in G6, and AcMYB20 was clustered with AtMYB20 in G31, while AcMYB70, AcMYB73 and AcMYB77 was clustered with AtMYB70 and AtMYB73 in G22. Generally, the exon-intron structure analysis of the *AcMYB* genes supported their phylogenetic relationships.

To investigate the structural diversity and predicted functions of the AcMYBs protein, a total of 10 conserved motifs in the AcMYBs were identified by the MEME software and subsequently annotated with InterProScan (see Supplementary Table S2). Of which, motif 2, 3 and 4 constituted the conserved R2 and R3 domain of ‘W-(X_19_)-W-(X_19_)–W -…-F/I-(X_18_)-W-(X_18_)-W-’. Motif 1, 6 and 9 were also identified as MYB domains, while other motifs (5, 7, 8 and 10) were of function unknown. Notably, all the AcMYBs had a MYB domain, and proteins of each AcMYBs subfamily shared some common motifs (Fig. 2C). Most of the MYB proteins in subfamily G9-1, G37-1, G5, and G18 contained motifs 1, 2, 3, 4, and 7; in subfamilies G6, G12, G14, G16, G18, G19, G20-1 and G20-2 contained motifs 1, 2, 3, and 4; in subfamily G10, G13, G24, G36, G9-2, G33, G37-2, and G31 contained motifs 1, 2, 3, 4, and 9; in subfamilies G21, G22, G23, G25, and G28 contained motifs 1, 2, 3, and 6; and in subfamily G27 contained motif 1, 2, 6, and 10 (Fig. 2C). Motifs 1 and 2 were found across the AcMYB gene families, while motif 10 and motif 8 are only present in subfamilies G27 and G23, respectively. Some of the genes present in the same group did encode proteins that differed in shared motifs. For instance, in G1 AcMYB30 had two additional motifs, motifs 4 and 7, compared to AcMYB10; and AcMYB82 lack motif 3 in G6. In general, most of the MYB genes clustered in the same subfamily showed similar motif characteristics with each other, implying that members in the same subfamily have a similar function.

### Expression profiles of *AcMYB* genes during fruit development

**Figure 3:**
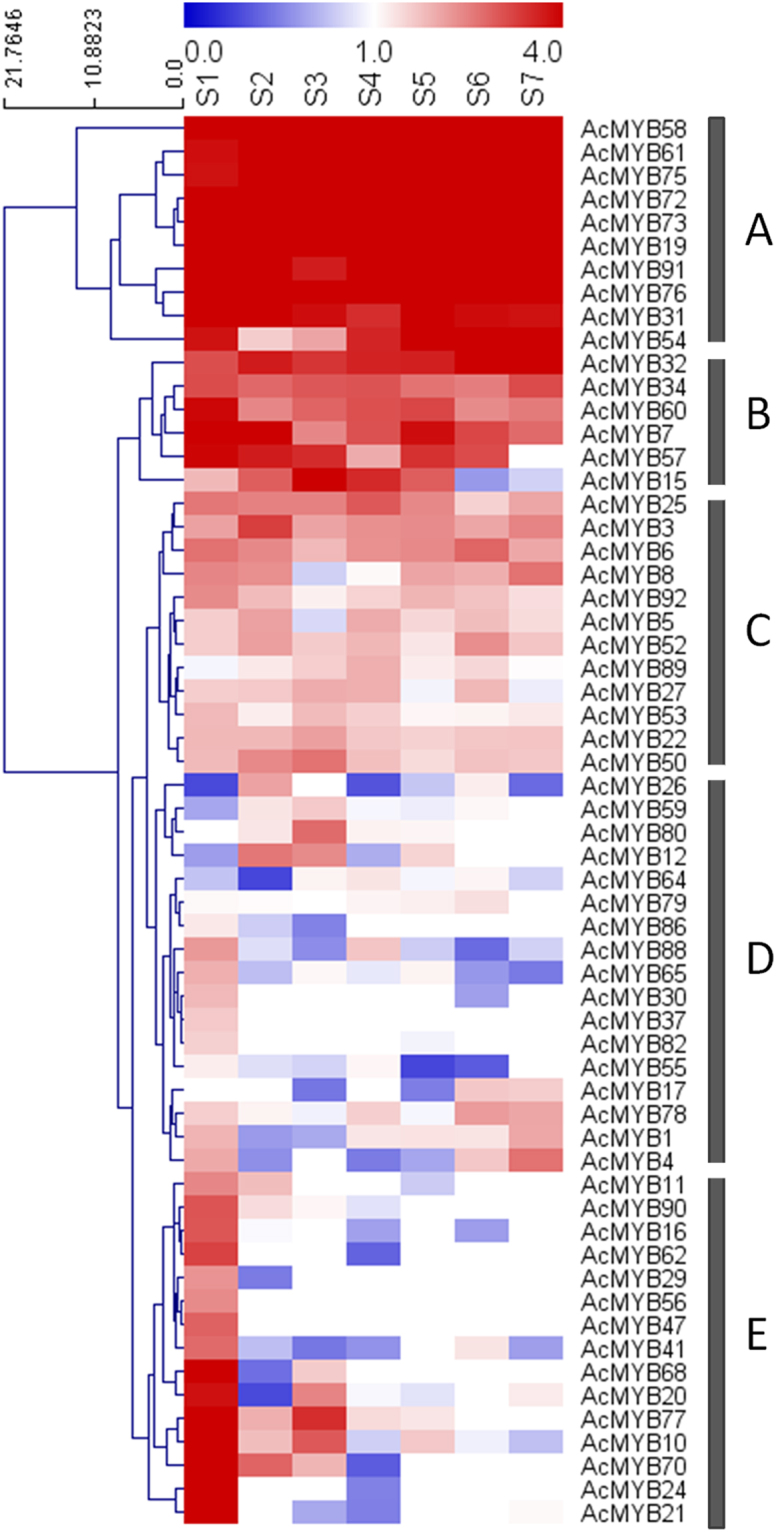
Expression profiles of *AcMYB* genes during different developmental stages of kiwifruit. Log_2_FPKM values were used to generate the heatmap with Hierarchical Clustering (complete linkage method, Euclidean distance) through software MeV (v. 4.8.1). Expression levels are represented by a color scale ranging from saturated blue for log_2_FPKM = 0 to saturated red for log_2_FPKM = 4. Seven stages of fruit development are shown (S1–S7).

To seek insights into the roles of *AcMYB* genes in kiwifruit development, the transcriptome data from fruit in the S1 to S7 stages was further investigated in this study. Transcripts were present for 60 *AcMYB* genes across the different development stages, but were not found in the RNA-seq libraries for the rest of the 33 *AcMYB* genes (Fig. 3; see Supplementary Table S3). Ten of the *AcMYB* genes (*AcMYB58*, *−61*, *−75*, *−72*, *−73*, *−19*, *−91*, *−76*, *−31*, *−54*; sorted into group A) exhibited high transcriptional abundance (FPKM value >16) during each of the 7 developmental stages, indicating key roles for these genes in fruit development. Genes in groups B and C showed decreasing expression levels from S1 to S7. Specially, *AcMYB57* and *AcMYB15* were not present at later stages of kiwifruit development. *AcMYB5*, *AcMYB52*, *AcMYB89*, *AcMYB27*, and *AcMYB53* had low transcript levels at S5. S5 is the stage with the highest anthocyanin accumulation in kiwifruit^32^, suggesting that these 5 *AcMYBs* may not be directly committed to anthocyanin biosynthesis. There were 17 *AcMYBs* in group D that overall were present at low levels throughout fruit development. For example, transcripts for *AcMYB26*, *AcMYB59*, *AcMYB12*, and *AcMYB64* were almost absent at S1, while the others had lower transcript levels at other stages. The 15 genes (*AcMYB-11*, *−90*, *−16*, *−62*, *−29*, *−56*, *−47*, *−41*, *−68*, *−20*, *−77*, *−10*, *−70*, *−24*, *−21*) in group E shared a pattern of the highest transcriptional levels (FPKM value >16) only at S1, with expression dropping across the other six stages. The transcript levels of these genes were higher at S1 than those in group D. The transcript levels of the newly identified genes *AcMYB75* and *AcMYB73* were high across all of fruit development, hinting that these genes might be involved in fruit development and formation of qualities such as flavor, color and taste. On the other hand, *AcMYB20*, *AcMYB70*, and *AcMYB77* in group E might specifically relate to blooming and preliminary development of fruit. For this comparison of the expression profiles of the *AcMYB* genes at different developmental stages, it can be concluded that *AcMYB* members play differential roles in fruit development.

### Molecular characterization of *AcMYB75*

**Figure 4:**
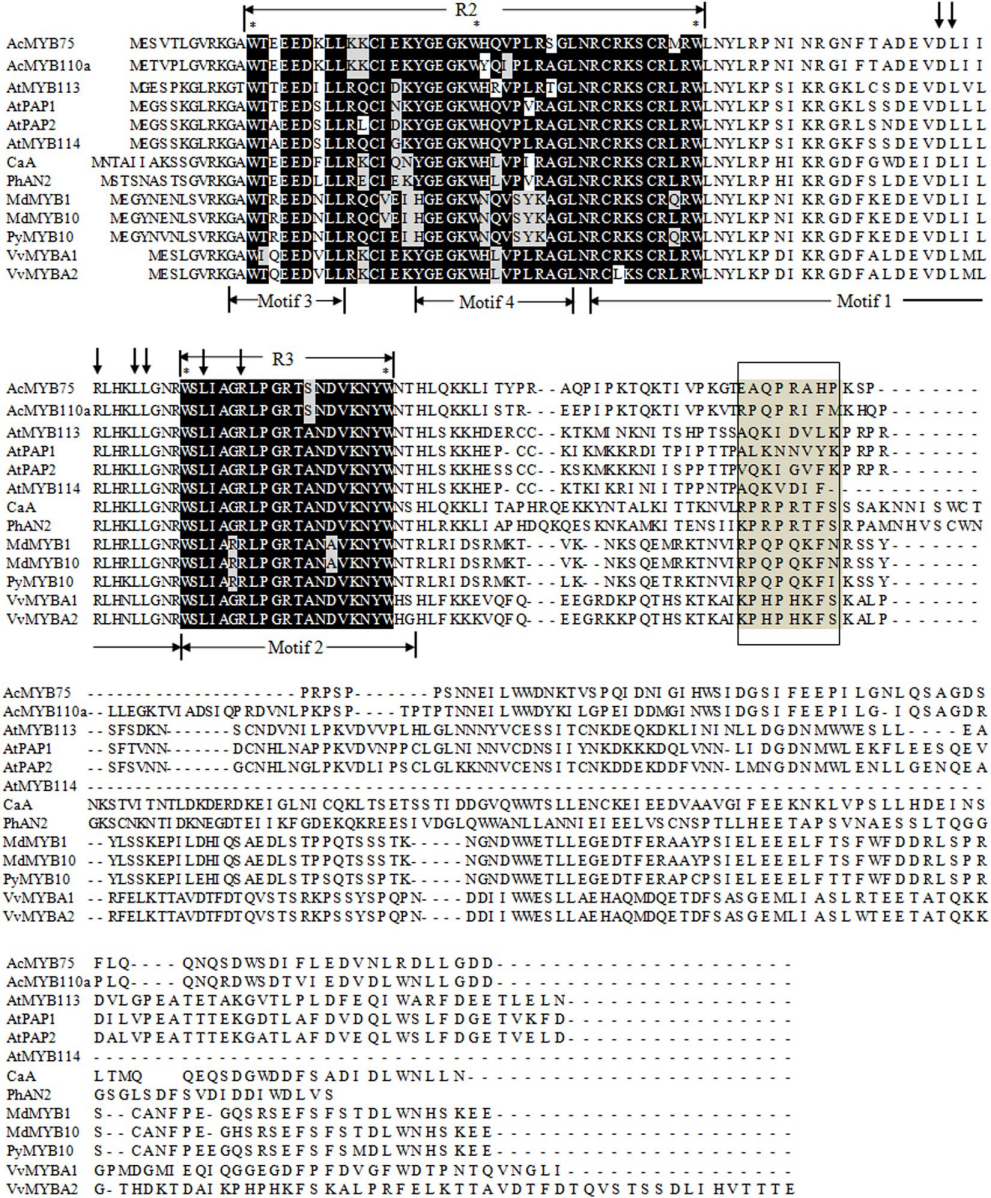
Alignment of AcMYB75 and its phylogenetic orthologs from other species Black and gray shadings indicate conserved amino acid residues. Horizontal arrows represent the R domains. Asterisks indicate the typical conserved amino acid in R domain. Vertical arrows indicate the motif ([DE]Lx_2_[RK]x_3_Lx_6_Lx_3_R) in the R2R3 domain, which allows interaction with a bHLH partner. The lower horizontal arrows represent the motifs from Fig. 2. The box indicates the motif typical of anthocyanin-related regulators. GenBank accession numbers are as follows: VvMYBA1 (*Vitis vinifera*, AGH68552.1), VvMYBA2 (*Vitis vinifera*, BAD18978.1), MdMYB10 (*Malus domestica*, ABB84753.1), MdMYB1 (*Malus domestica*, ABK58136.1), CaA (*Capsicum annuum*, CAE75745.1), PyMYB10 (*Pyrus pyrifolia* var. culta, ADN26574.1), AcMYB110a (*Actinidia chinensis*, AHY00342), AtPAP1 (*Arabidopsis thaliana*, Q9FE25.1), AtPAP2 (*Arabidopsis thaliana*, Q9ZTC3.1), AtMYB113 (*Arabidopsis thaliana*, Q9FNV9.1) and AtMYB114 (*Arabidopsis thaliana*, Q9FNV8.1) protein sequences.

Since AcMYB75 was clustered with AtMYB75 (PAP1) and AtMYB90 (PAP2) (Fig. 1, G6) that was verified to regulate the accumulation of anthocyanin in *Arabidopsis thaliana* and *AcMYB75* also had the higher expression levels during anthocyanin accumulation in kiwifruit, *AcMYB75* was studied further for its regulation on anthocyanin biosynthesis in kiwifruit. The full-length cDNA of *AcMYB75* was cloned and sequenced. The result verified that *AcMYB75* had 666 nucleotides and contained an open reading frame (ORF) encoding a deduced protein of 221 amino acids. The deduced AcMYB75 protein contained R2 (motif 3 and 4) and R3 (motif 2) MYB domains (horizontal arrows) and a motif typical of anthocyanin-related regulator (box)^27^ (Fig. 4). The amino acid of [DE]Lx_2_[RK]x_3_Lx_6_Lx_3_R (vertical arrows) in motif 1 and motif 2 (R3) were related to the bHLH interaction^35^. There are 49 amino acids different between AcMYB75 and AcMYB110a, four of which are in the conserved R2, while those in the R3 MYB domains are completely the same. In the 8 amino acid motif of anthocyanin-related regulators, only three of the residues (Q, P and R) were the same between AcMYB75 and AcMYB110a. Our phylogenetic tree showed the bootstrap value of AcMYB75 and other MYBs regulating anthocyanin accumulation, including PAP1 and PAP2 from *Arabidopsis* ^36^, AcMYB110a from kiwifruit^27^, PyMYB10 from *Pyrus pyrifolia* var. culta^25^ and VvMYBA from *Vitis vinifera* ^37^, of which 99% supported the clustering of AcMYB75 and AcMYB110a (Supplementary Fig. S2).

**Figure 5:**
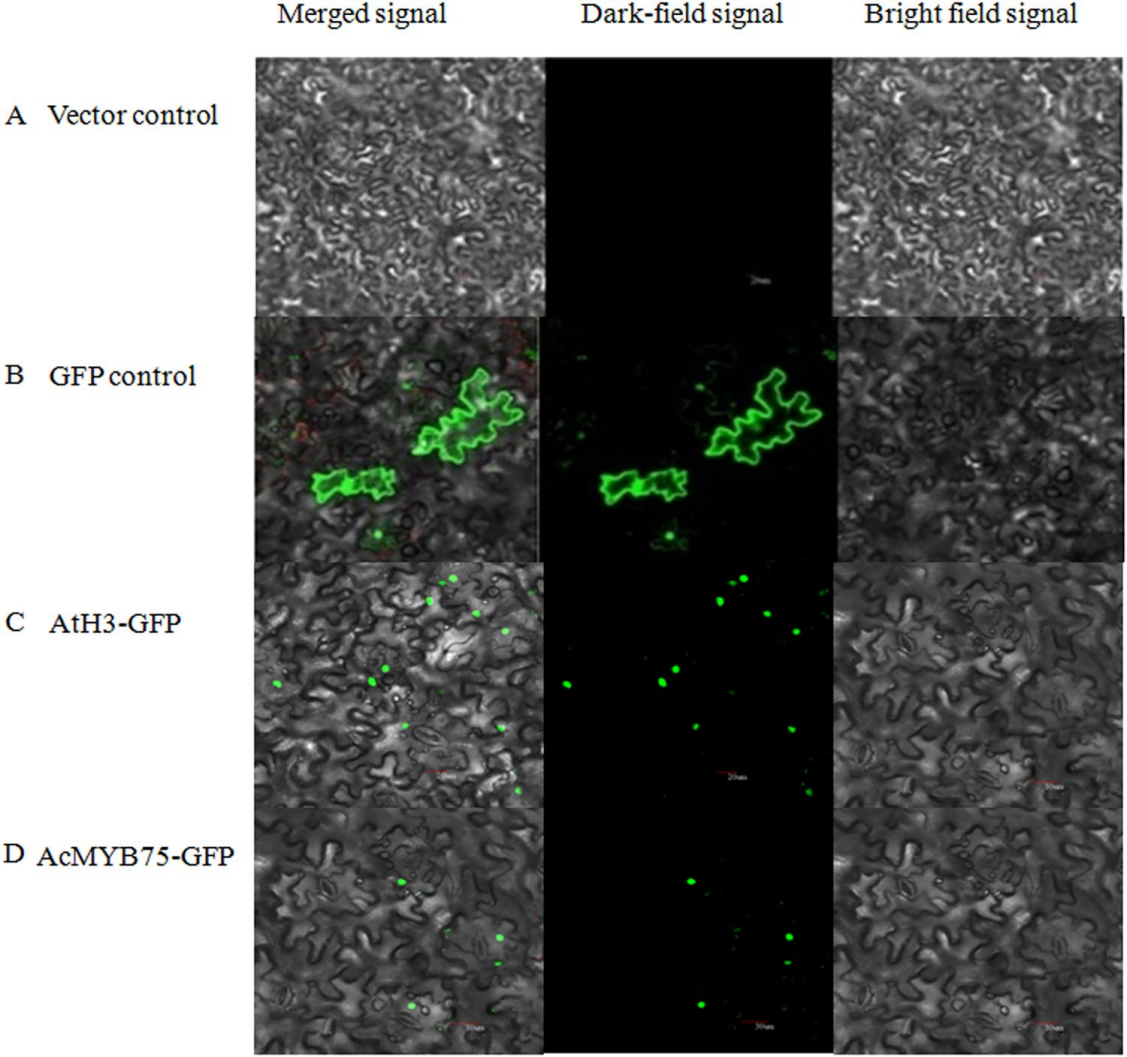
Sub-cellular localization of *AcMYB75* AcMYB75 was transiently expressed in tobacco epidermal cells. The tobacco cells were observed under a confocal microscope (Olympus FV1000, Japan). (**A**) vector control; (**B**) GFP control; (**C**) nuclear-localized GFP control; (**D**) AcMYB75-GFP.

Using fluorescence microscopy to study the subcellular localization of *AcMYB75*, vector-only constructs were not detected in cytoplasm or nucleus (Fig. 5A), while vector-GFP constructs were distributed in both cytoplasm and nucleus (Fig. 5B). The positive control AtH3-GFP showed signal specifically in nuclei due to nuclear targeting of Histone 3 protein (Fig. 5C). AcMYB75-GFP fusion proteins were also found localized exclusively in the nuclei, suggesting that the AcMYB75 protein had nuclear targeting sequences (Fig. 5D).

### Transcriptional activation and DNA binding of *AcMYB75*

**Figure 6:**
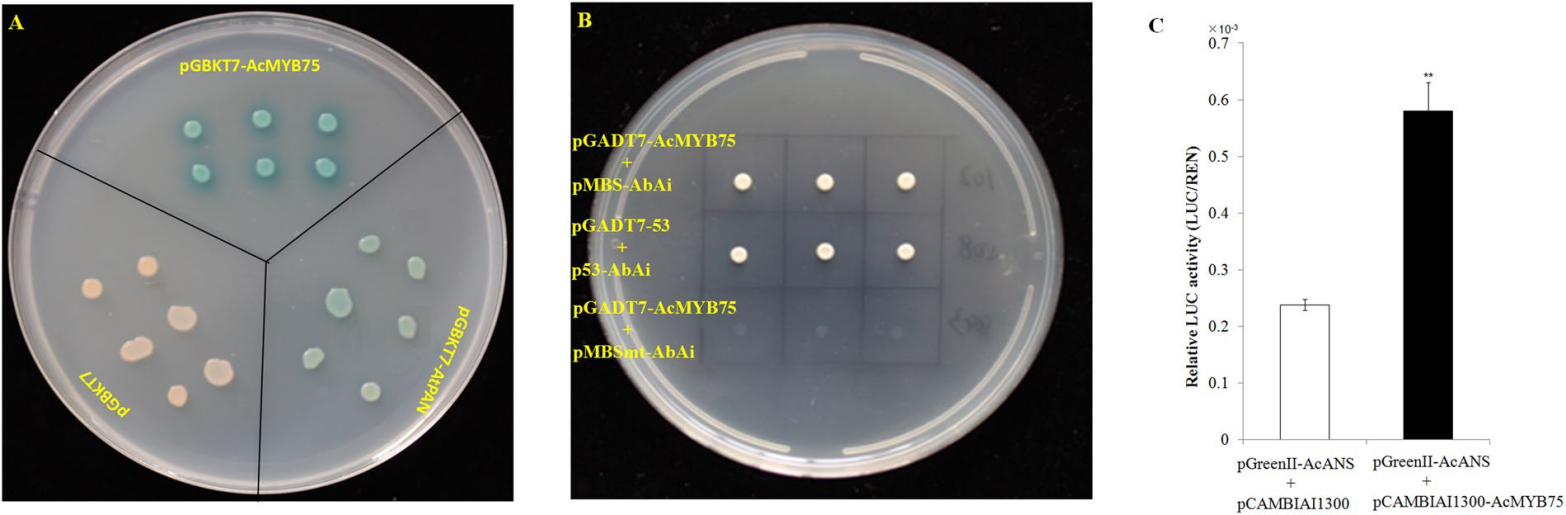
Transactivation and binding specificity of *AcMYB75* (**A**) Yeast cells carrying LacZ reporter gene were grown in SD/-Trp medium at 30 °C for 3 d. X-α-gal was used for selection. Six replications. (**B**) Yeast cells were grown in SD/-Ura selective medium containing 200 ng ml^−1^ AbA for 3 d at 30 °C. (**C**) LUC/REN ratio from transient expression analysis. Values are means of eight replicates. Bars indicate ± standard error. **P < 0.01.

The transcriptional activation and DNA binding characteristics of AcMYB75 as transcription factor were verified through yeast one-hybrid system. The AcMYB75 protein significantly activated *LacZ* expression under control of G1 promoter through interaction with GAL4 binding domain (Fig. 6A). This verified that *AcMYB75* possessed a transcription activation domain (AD). In addition, we found only yeast clones expressing either AcMYB75 and pMBS-AbAi (MYB binding sequences (MBS) cloned into the bait vector pAbAi) or the positive controls could grow on SD/-Leu selective medium containing AbA (Fig. 6B). This result suggested that AcMYB75 can bind with the MBS of the *ANS* promoter from kiwifruit through the DNA binding domain and that the DNA binding is sequence specific, as the mutations of three ‘TT’ pairs to three ‘CC’ pairs in the MBS disrupted the interaction between AcMYB75 and the target promoter of *AcANS*.

Furthermore, we investigated the interaction between the *AcANS* promoter and *AcMYB75 in vivo* through the dual-luciferase reporter assay system. The promoter is cloned as a transcriptional fusion with firefly luciferase gene (LUC) and LUC activity is expected to increase when TFs bind the promoter. The same construct contains a luciferase gene from *Renilla* (REN) driven by a 35 S promoter that served as a control for normalization. Activity is identified as a ratio of LUC to REN-luc activity. It showed that the *AcMYB75* cDNAs was able to induce an approximately 2-fold increase in luciferase (LUC) enzyme activity when co-infiltrated with construct containing the AcANS promoter fused to the firefly LUC gene (Fig. 6C). The result suggested that *AcMYB75* could cause trans-activation in kiwifruit.

### Anthocyanin biosynthesis in transgenic *Arabidopsis* over-expressing *AcMYB75*

**Figure 7:**
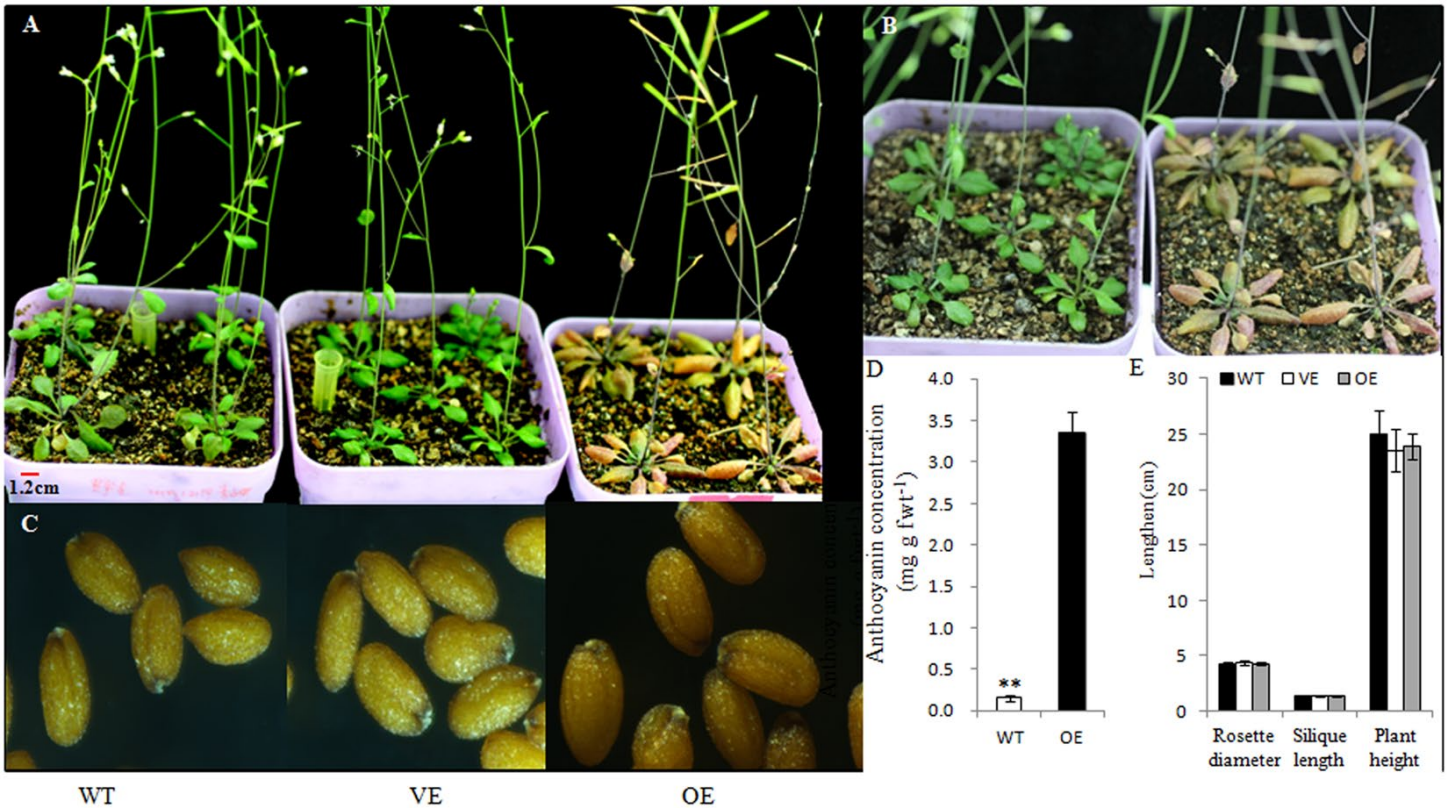
Phenotype of transgenic *Arabidopsis* with over-expressed *AcMYB75* (**A**) Phenotype of *Arabidopsis* plants. WT: wild type, VE: vector only, OE: *AcMYB10* over expressed. (**B**) Enlarged rosette picture of *AcMYB10-OE Arabidopsis*. Left: WT, Right: OE. (**C**) Phenotype of *Arabidopsis* seeds under stereoscopic microscope (50×) (**D**) Concentration of anthocyanin in leaves of OE and WT *Arabidopsis* plants. **P < 0.01. Values are means of five replicates. (**E**) Measurement of rosette diameter, silique length and plant height. Bars indicate ± standard error.

*AcMYB75* over-expressing (OE) *Arabidopsis* plants had purple leaves, especially leaves in the rosette compared to those from wild type (WT) and vector only (VE) plants (Fig. 7A). Leaves in the rosette in OE plants appeared a little bit curved downward compared to the leaves from WT (Fig. 7B). The color and size of seeds from OE were similar to those from WT and VE plants (Fig. 7C). The concentrations of anthocyanins in OE leaves was significantly increased compared to that of WT plants (3.35 vs 0.154 mg g fwt^−1^) (Fig. 7D), while the difference was hardly detectable between WT and VE plants (data not shown). Other phenotypes were similar among OE, WT and VE plants, such as rosette diameter, plant height, and siliques length (Fig. 7E).

**Figure 8:**
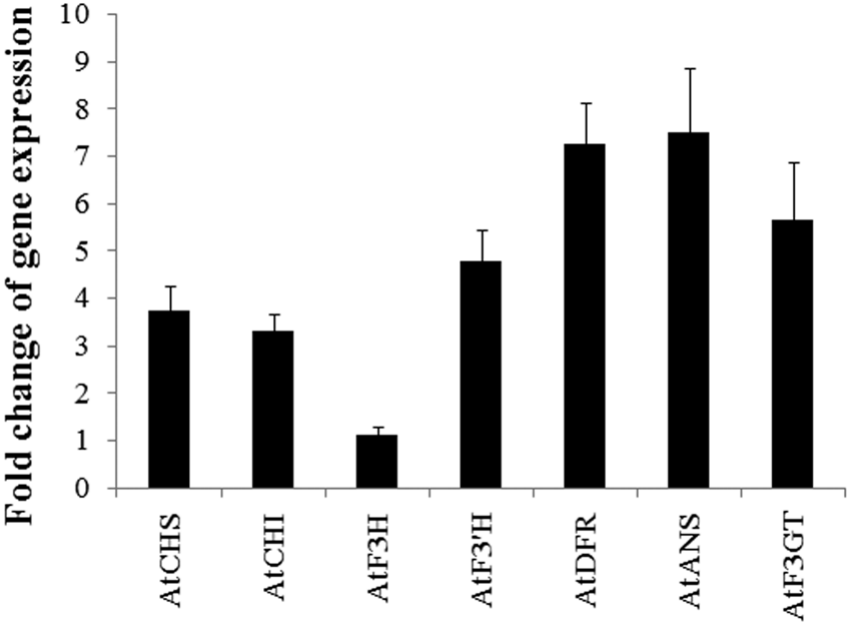
Expression profiles of genes in OE *Arabidopsis* leaves compared with WT. Values are means of five replicates and bars indicate ± standard error. The relative expression levels of genes were calculated by using the 2^−ΔΔCt^ method, and the fold change of gene expression is the ratio of gene expression in OE plants to that of the same gene in WT plants. CHS, chalcone synthase; CHI, chalcone isomerase; F3H, flavanone 3-hydroxylase; F3′H, flavonoid 3′-hydroxylase; DFR, dihydroflavonol 4-reductase; ANS, anthocyanin synthase; F3GT, anthocyanin 3-O-glucosyltransferase.

We also examined the expression of early (*CHS*, *CHI*, *F3H*, and *F3′H*) and late (*DFR*, *ANS* and *F3GT*) flavonoid biosynthetic genes. As a whole, the results showed that the expression levels of these genes were up-regulated in *AcMYB75* over-expressing transgenic *Arabidopsis* plant. Particularly, the expression levels of *AtANS* and *AtF3GT* that determine the last steps of anthocyanin biosynthesis were about 7 and 5 fold higher in OE plants than in WT plants (Fig. 8). Together, these results suggest that *AcMYB75* be a candidate transcription factor regulating anthocyanin biosynthesis in kiwifruit.

## Discussion

Kiwifruit (*Actinidia chinensis*) originates from China and has become an important commercial horticultural crop due to its pleasant flavor and nutritional components benefiting human health^38^. For example, the red-fleshed Hongyang kiwifruit, derived from *A*. *chinenesis* var. *chinensis* ^39^, is becoming a favorite of consumers, breeders and growers due to its unique phenotype, excellent agricultural characteristics, and high premium price at market. However, research progress on kiwifruit was relatively slow compared to other important horticultural crops, such as grape, apple or tomato. A comprehensive analysis of the R2R3 *AcMYB* gene family has yet to be reported, and the functions of most R2R3 *AcMYBs* are still unknown. In this study, we comprehensively analyzed the R2R3 *AcMYB* family, by looking at their phylogenetic relationships, gene structures, protein features, and expression profiles during fruit development.

The number of R2R3 *AcMYB* genes identified in kiwifruit (93) was lower than in *Pyrus bretschneideri* (105)^7^, *Glycine max* (244)^30^, *Malus pumila Mill*. (222)^40^, *Arabidopsis thaliana* (126)^8^, *Zea mays* (157)^41^, and *Solanum lycopersicum* (127)^42^ but higher than in *Cucumis sativus* (55)^43^ and *Orzya*. *sativa* (88)^28^. The phylogenetic relationship of *MYB*s among kiwifruit, *Arabidopsis* and tomato indicated that the three species displayed both conserved and specialty evolution of *MYB* members, particularly in kiwifruit and tomato. As examples, group C12 contained genes only from kiwifruit and tomato; group C1 had no genes from kiwifruit; and groups C3, C8 and C17 had more *MYB* genes than other groups. These results suggested that species-specific MYB genes were either acquired in kiwifruit or lost in *Arabidopsis* lineages after divergence from their most recent common ancestor.

Notably, most genes within the same group generally share similar exon-intron patterns in our genes structure analysis. Differences in exon-intron patterns, lengths or presence of proteins motifs may suggest hints at the function of these genes and how they evolved.

Through detailed bioinformatics analysis of genome and transcriptomic data, 5 R2R3-MYB genes were newly identified and annotated. It is well known that R2R3-MYB genes are extensively involved in plant growth and development^44^. Functional analysis of MYB transcription factors has been performed in *A*. *thaliana* and other species^45,46^. *AtMYB77* and *AtMYB44* are associated with fruit development, stress response, and also auxin and abscisic acid signal transduction in *Arabidopsis* ^47,48^, suggesting that *AcMYB70*, *AcMYB73*, and *AcMYB77*, which closely clustered with *AtMYB77* and *AtMYB44* in the same subfamily (Fig. 1), may have similar functions. *AcMYB20* and *SlMYB20* might also be related to stress response like *AtMYB20* ^49^. *AcMYB75*, which clustered with *AtMYB75*/*PAP1* and *AtMYB90*/*PAP2* that regulate anthocyanins biosynthesis in *Arabidopsis* ^34,50,51^, may also function in anthocyanin accumulation regulation in kiwifruit.

Transcript profiles can provide important clues regarding gene functions. *AcMYB20*, *AcMYB70* and *AcMYB77* may have roles in the development of young kiwifruit due to their expression levels at S1. The transcripts of *AcMYB58*, *AcMYB61*, *AcMYB73* and *AcMYB75* were high during the stages in kiwifruit development that have high anthocyanin accumulation, implying that they could be necessary for both fruit development and anthocyanin biosynthesis.

In this study, we provided multiple lines of evidence such as sub-cellular localizaton experiment in tobacco, promoter transactivation assay in yeast and plant cell, and over-expression in *Arabidopsis* that support the role of *AcMYB75* as a transcription factor in the anthocyanin biosynthetic pathway in plants. R2R3-MYB TFs affect anthocyanin biosynthesis through regulation of flavonoid/phenylpropanoid biosynthetic genes. For instance, *AtMYB75* regulates enzymes that convert phenylalanine into naringenin chalcone including PAL, C4H, 4CL and CHS, and also DFR enzyme catalyzing dihydrokaempferol into leucopelargonidin^52^. In peach, *MYB10*.*1* and *MYB10*.*3* induce anthocyanin production by up-regulating UFGT and DFR genes in transgenic tobacco^53^. *MYB110a* plays an important role in the regulation of *F3GT1* expression in the red flowers of kiwifruit^27^. Our results showed that over-expressed *AcMYB75* also functioned in a heterologous system and could positively up-regulate the expression levels of anthocyanin biosynthetic genes in *Arabidopsis* plants. However, the constitutive expression of *AcMYB75* in transformed *Arabidopsis* plants resulted in incomplete purple leaves, and not the extreme red phenotype produced when *AtMYB75* is over-expressed^34^. This phenomenon has been shown in some previous researches^17,54^. Since a general WD-repeat/MYB/bHLH model for regulation of the anthocyanin biosynthetic pathway was found to operate in many plant species including snapdragon, strawberry, petunia, *Arabidopsis thaliana* (Morita *et al*., 2006; Quattrocchio *et al*., 1999; Schwinn *et al*., 2006; Spelt *et al*., 2000; de Vetten *et al*., 1997; Zhang *et al*., 2003, Schaart *et al*., 2013; Gonzalez *et al*., 2008), we investigated the interaction between AcMYB75 and bHLH type transcription factors from *Arabidopsis* through the yeast two-hybrid system. The result showed that AcMYB75 had interaction with N-terminal domains of TT8 snd GL3 (Supplementary Fig. S3). The phenotype of the AcMYB75 over-expressed *Arabidopsis* plants might due to other subtle and complex regulations including interaction with flavonoid structural gene promoters or posttranscriptional or posttranslational regulation.

Furthermore, the MYB binding sites in kiwifruit should be verified further in the future. As reported, TAACTG was the MYB recognition sequence of *MYB2* in *Arabidopsis* ^55^. The MBSI (aaaAaaC(G/C)GTTA) and MBSII (aaaAGTTAGTTA) were the binding sites of *Ph3* in *Petunia* hybrid and involved in flavonoid biosynthetic genes regulation^56^. We also speculated that the TT residue in the MBS might be related to the binding of *AcMYB75* with *AcANS*. However, the whole or key MYB binding sequences in flavonoid biosynthetic genes in kiwifruit should be verified furthermore.

## Methods

### Plant materials

Red-fleshed Hongyang kiwifruit (*A*. *chinensis*) were collected from the Wuhan Botanical Garden, Hubei Province in 2015. Samples from seven stages of fruit development from 7 (S1), 70 (S2), 90 (S3), 100 (S4), 110 (S5), 120 (S6), and 140 (S7) days after anthesis were harvested according to the development and the anthocyanin accumulation of kiwifruit as our previous research^32^. Briefly, the highest anthocyanin is kiwifruit ovary at S1, but disappears at S2. During the expansion of kiwifruit, anthocyanin accumulation starts again at S3. The deepest red color appears from S4 to S5, but anthocyanin will lost a little from S6 to S7 when fruit matures.

### Transcriptome data

Transcriptome data from seven stages of fruit was obtained from our previous experiments^32^. Transcriptome analysis had deposited at DDBJ/EMBL/GenBank under the accession numbers GALZ00000000, GAMA00000000 and GAMB00000000as previously described and transcriptome analysis was performed as previously described^57,58^. The expression of R2R3 MYB genes was normalized and calculated as FPKM (Fragments Per Kilobase of exon per Million fragments mapped) using Cuffdiff (v2.1.1)^59^.

### Identification of R2R3 MYB genes in kiwifruit

To identify putative R2R3-MYB genes in kiwifruit across the kiwifruit genome, the MYB_DNA-binding (PF00249) family from PFam (http://pfam.sanger.ac.uk/) was used to query the kiwifruit protein sequences obtained from Kiwifruit Genome Database (http://bioinfo.bti.cornell.edu/cgi-bin/kiwi/home.cgi)^31^ by local HMM-based searches with E-values <0.01^60^. Other R2R3-MYB protein sequences from *Arabidopsis* and tomato (*Solanum lycopersicum*) were included from UniPort (http://www.uniprot.org/) and Phytozome (http://www.phytozome.net/slycopersicum.php)^42^. Subsequent, BLAST searches were performed to check the predicted kiwifruit R2R3 MYBs with all the *Arabidopsis* and tomato R2R3 MYBs as queries. In addition, pair-end reads from our transcriptome data set were used to identify novel MYB candidates as previously described^58^. Briefly, clean reads were mapped to kiwifruit genome using Tophat v2.0.10 allowing novel splice junctions identification (read mismatch = 1)^61^. Novel transcripts were identified by cufflink and cuffcompare in the Cufflinks v2.2.1^62^. For each transcript sequence, the coding regions and related protein sequences were predicted by TransDecoder v3.0.0 (http://transdecoder.github.io/). Finally, all candidate protein sequences were further examined by the CDD (http://www.ncbi.nlm.nih.gov/cdd/) (threshold = 0.01, maximum hits = 500) and PFAM (http://pfam.sanger.ac.uk/) (E-value = 1.0) databases.

### Phylogenetic and sequence analyses

Multiple sequence alignments were applied to confirm the conserved domains of predicted R2R3 AcMYB proteins. Additional, sequence alignments of the full-length R2R3 MYB proteins from kiwifruit, *Arabidopsis* and tomato were performed using ClustalX 2.0^63^. Based on the sequence alignments, a neighbor-joining phylogenetic tree was constructed using MEGA 6.0^64^ software with Poisson model and 1000 bootstrap replicates, and the R2R3-MYBs were assigned to subfamilies according to the previous classification in *Arabidopsis* ^8,33^.

The ExPASy proteomics server (http://expasy.org/) was employed to detect the molecular weight and isoelectric points of the R2R3 AcMYB proteins^65^. Exon-intron structures were predicted with Gene Structure Display Server (GSDS, http://gsds.cbi.pku.edu.cn/)^66^. The conserved motifs of kiwifruit MYB proteins were identified using the MEME program (http://meme.ncbr.net/meme/cgi-bin/meme.cgi), with optimum motif width ranging from 11 to 50 and the maximum number of motifs set to ten^67^. All identified motifs were annotated with interProScan (http://www.ebi.ac.uk/Tools/pfa/iprscan/). Hierarchical cluster analysis was performed through MeV software from the TM4 suite (http://www.tm4.org/mev.html)^68^.

### RNA extraction, cDNA synthesis and quantitative real-time RT-PCR (qRT-PCR) analysis

Total RNA was isolated using plant RNA kit (OMEGA, USA). First-strand cDNAs were synthesized using the FastQuant RT Kit (with gDNase) (TIANGEN, China), according to the manufacturer’s instructions, before use as templates for real-time PCR amplification.

The PCR mixture contained 1 μl of the cDNA template, 10 μl of 2 × SYBR^®^ Premix Ex Taq^TM^ (DRR041A, Takara), and 0.2 μM of the forward and reverse primers for each gene. Reactions were run on an Applied Biosystems StepOne Real-Time PCR system (Applied Biosystems, USA). The conditions for each PCR reaction were as following: 95 °C for 30 s, followed by 40 cycles of 5 s at 95 °C, 20 s at 58–62 °C depending on primer TM, and 20 s at 72 °C. At the end of each experiment, a melt-curve analysis was performed using the default parameters (15 s at 95 °C, 60 s at 55–95 °C in 0.3 °C increments, 15 s at 95 °C). The relative expression levels of the target genes were calculated by the 2^−ΔΔCt^ method. The β-actin 2 (At3g18780) and glyceraldehyde-3-phosphate dehydrogenase A (*GAPDH*) (At3g22650) genes were used as internal references to normalize the transcriptional levels of target genes. All genes involved in the flavonoid pathway in *Arabidopsis* ^36^ were chosen. Genes and primers are listed in Supplementary Table S4.

### Subcellular localization analysis

The full length cDNA of *AcMYB75* was amplified using the following primers: F: 5′-TGC TCT AGA ATG GAA AGT GTT ACT TTA GG-3′, R: 5′-CG GGA TCC ATC ATC ACC TAA GAG ATC CC-3′ and cloned into the plant expression vector of pCAMBIAI1300 under the control of CaMV35S promoter. The histone H3 acetyltransferase gene (*AtH3*, AT3G14980) was used as a positive reference. *Agrobacterium tumefaciens*, LAB4404 with the plasmids of *CaMV*35S::*AcMYB75*-*GFP*, *CaMV*35S::*AtH3*-*GFP* or *CaMV*35S (vector only) were infiltrated into tobacco (*Nicotiana benthamiana*) epidermal cells, respectively. Infiltrations were performed according to the methods of Li *et al*.^68^. After 3–5 d of incubation at room temperature, the tobacco cells were observed under a confocal microscope (Olympus FV1000, Japan).

### Transactivation by AcMYB75

The GAL4 one-hybrid system was used for identifying the transactivation of AcMYB75 in Y187 yeast (*Saccharomyces cerevisiae* Y187:ura3–52, trp 1–901, Ura3::GALUAS-Gall TATA-LacZ) (Clontech, USA). The full-length of *AcMYB75* cDNA was fused with the GAL4 DNA-binding domain to generate pGBKT7-AcMYB75 through *EcoRI*/*BamHI*. The gene PERIANTHIA (*PAN*), reported as a bZIP transcription factor, was used as the positive control^69^. The pGBKT7-*AcMYB75*, pGBKT7-AtPAN and pGBKT7 constructs were transformed by PEG/LiAc method into yeast strain Y187 according to the yeast transformation system 2 user manual (Clontech, USA). The reporter genes *LacZ* is controlled by the G1 promoter (the GAL4 binding domain responding cis-element). The Y187 transformants were selected on SD/-Trp at 30 °C for 3 d. X-α-gal was used for the confirmation of transcriptional activation.

### DNA-binding domain verification in AcMYB75

The Matchmaker Gold Yeast One-Hybrid Library Screening System (Clontech, USA) employing strong selective power of Aureobasidin A (AbA) resistance to produce screens with very low backgrounds was used for the identifying of the DNA-binding domain in AcMYB75. Resistance to AbA is conferred by the AbAr gene (*AUR-1C*) which is the reporter on the bait vector pAbAi. Activation of the AbA resistance gene (AbAr) occurs, if the binding domain of transcription factors bind to the bait sequence. Three tandem repeats of the putative MYB binding sequence (MBS) from anthocyanin synthetase (ANS) (Achn361621) promoter (273 bp before ATG). promoter were synthesized (Sangon Biotech, China) (5′-TGGATCTCTATCTCACGACGG**TT**AGACCACCTCGTGCTTGTCTCACGACGG**TT**AGACCACCTCGTGCTTGTCTCACGACGG**TT**AGACCACCTCGTGCTTGTGCCATCCAG-3′) and cloned into the *SacI*/*XhoI* on the pAbAi vector under the yeast iso-1-cytochrome C minimal promoter as the bait (pMBS-AbAi). The full length of AcMYB75 was fused with the GAL4 transcription activation domain to generate pGADT7-AcMYB75 through *EcoRI*/*BamHI* under the constitutively active ADH1 promoter (*P*_*ADH1*_) as the prey.

The sequence with three ‘TT’ pairs mutated to three ‘CC’ pairs at positions 22–23, 52–53, and 82–83, respectively, designated as pMBSmt-AbAi, was used as negative control. The plasmids pMBS-AbAi, pMBSmt-AbAi or p53-AbAi were integrated into yeast strain Y1HGold by homologous recombination to construct the bait yeast according to the user manual. The concentration of 200 ng ml^−1^ AbA was determined to suppress basal expression of the bait construct. Subsequently, the constructed prey plasmids of pGADT7-AcMYB75 and pGADT7-53 were individually transformed into the corresponding bait yeast according to the yeast transformation system 2 user manual (Clontech, USA). The recombinant Y1HGold yeast cells with bait and prey plasmids were selected on SD/-Ura medium with 200 ng ml^−1^ AbA, at 30 °C for 3 d.

### Dual luciferase assay of transiently transformed tobacco leaves

The *ANS* promoter was insterted into the plasmid of pGreenII^70^ to construct the plasmid of pGreenII-AcANS. The *Agrobacterium tumefaciens* strain LBA4404 cultures transformed with pGreenII-AcANS were mixed with the *Agrobacterium tumefaciens* cultures transformed with constructed plasmids of pCAMBIAI1300*-*AcMYB75 according to v/v (1:5) for co-infiltration. Nicotiana plants were grown under glasshouse conditions under natural light with 16 h daylight for transient transformation. The *Agrobacterium* mixtures were injected into tobacco leaves according to Li’s method^68^.

Three days after infiltration, 1 cm leaf discs (six technical replicates) were collected and the activity of luciferase and REN-Luc were measured according to Dual-Luciferase Reporter Assay System manufacture’s manual (Promega, USA). The binding ability of AcMYB75 to the promoter of AcANS were represented by LUC/REN (ratio of LUC to REN-luc).

### Yeast two-hybrid Screening

The yeast two-hybrid screening was used to verify the interaction between AcMYB75 and endogenous bHLH transcriptors from *Arabidiopsis*, *AtTT8* (At4g09820) and *AtGL3* (At5g41315)^71,72^. The two DNA binding domains of *AtTT8* and *AtGL3* were cloned into pGBKT7 vector to generate pGBKT7-AtTT8-1, pGBKT7-AtTT8-2, pGBKT7-AtGL3-1, and pGBKT7-AtGL3-2, respectively, as the binding domain plasmids. The yeast strain AH109 with four report genes of *LacZ*, *HIS3*, *ADE2*, *MEL1* under the control of *GAL1*, *GAL2* and *MEL1* promoters was used for the screening^73^. The constructed activation domain plasmids (pGADT7-AcMYB75) and four binding domain plasmids (pGBKT7-AtTT8-1, pGBKT7-AtTT8-2, pGBKT7-AtGL3-1, and pGBKT7-AtGL3-2) were co-transformed into AH109, respectively, according to the yeast transformation system 2 user manual (Clontech, USA). The transformants were selected on SD/-Leu/-Trp at 30 °C for 3 d. and then the positive transformants were again selected on SD/-Ade/-His/-Leu/-Trp at 30 °C for 3-5 d. The positive transformants from SD/-Ade/-His/-Leu/-Trp were ultimately selected through X-α-gal with SD/-Ade/-His/-Leu/-Trp again at 30 °C for 3–5 d.

### Generation of transgenic *Arabidopsis*

*Arabidopsis thaliana* ecotype Columbia (Col-0) plants were grown in growth rooms at 22 °C in long (16-hr of light) or short (9-hr of light) days. The constructed plasmids of *CaMV35S::AcMYB75* for subcullular localization analysis were electroporated into *Agrobacterium tumefaciens* LBA4404 and then transformed into *A*. *thaliana* plants by the floral dipping method^74^. Four independent single copy insert lines (1–6, 7–4, 6–18 and 10–30) were taken to the third generation for use in experiments. Seeds of individual self-fertilized T_2_ lines were collected and single-copy insertion lines were selected based on a Mendelian segregation ratio. All healthy leaf material and seeds of T_2_ lines were collected from individual plants and immediately frozen in liquid nitrogen and stored at −80 °C.

### Anthocyanin measurement

Samples (1 g for leaves) were ground in liquid nitrogen before anthocyanin extraction using 5:1 (v/w) ethanol/H_2_O/acetic acid (80:20:1 v/v/v) in an Ultra-Turrax homogenizer for 30 minutes and extraction overnight at 4 °C. Anthocyanin extraction and detection were as described^32,75^. Anthocyanin concentration was calculated from the peak area at 530 nm as cyanidin 3-O-glucoside.

## Electronic supplementary material

Supplementary Tables

Supplementary Figures
