# Supplementary material for: Kiwifruit R2R3-MYB transcription factors and contribution of the novel AcMYB75 to red kiwifruit anthocyanin biosynthesis
Source: Sci Rep. 2017 Dec 4;7:16861. doi: 10.1038/s41598-017-16905-1 (PMC5715094; doi:10.1038/s41598-017-16905-1)
Supplement: Supplementary file 1 — Supplementary Tables [file 41598_2017_16905_MOESM1_ESM.pdf]

Supplementary materials

Kiwifruit R2R3-MYB transcription factors and contribution of the novel *AcMYB75* to red kiwifruit anthocyanin biosynthesis

Wenbin Li[1 †], Zehong Ding[1 †], Mengbin Ruan[1], Xiaolin Yu[1], Ming Peng[1*], Yifei Liu[2*]

Supplementary Table S1. Characteristics of R2R3-MYBs in kiwifruit

No.	Ac.myb	Ac.ID	AA_length	PI	MW/kD
1	AcMYB1	Achn013291	431	6.41	46.49
2	AcMYB2	Achn064821	247	6.48	28.13
3	AcMYB3	Achn294101	224	8.97	25.62
4	AcMYB4	Achn020361	241	8.98	27
5	AcMYB5	Achn148821	287	8.78	32.2
6	AcMYB6	Achn372141	277	8.76	30.6
7	AcMYB7	Achn322351	225	9.08	25.71
8	AcMYB8	Achn143561	228	9.13	25.97
9	AcMYB9	Achn047241	282	6.83	31.58
10	AcMYB10	Achn234881	275	6.61	30.76
11	AcMYB11	Achn358591	240	9.42	27.92
12	AcMYB12	Achn013391	207	9.33	23.28
13	AcMYB13	Achn172901	240	8.14	27.34
14	AcMYB14	Achn081181	290	5.86	32.67
15	AcMYB15	Achn382471	261	5.43	30.42
16	AcMYB16	Achn377521	295	8.79	33.06
17	AcMYB17	Achn197141	292	8.59	32.73
18	AcMYB18	Achn027081	277	8.67	31.42
19	AcMYB19	Achn358581	403	6.55	46.36
20	AcMYB20	Gene.3370	297	4.96	33.31
21	AcMYB21	Achn297221	217	6.38	24.9
22	AcMYB22	Achn225741	1010	5.09	110.79
23	AcMYB23	Achn295821	488	8.76	54.9
24	AcMYB24	Achn389471	214	5.92	24.48
25	AcMYB25	Achn055151	409	5.88	44.87
26	AcMYB26	Achn198731	311	6.92	35.1
27	AcMYB27	Achn211821	401	5.75	43.98
28	AcMYB28	Achn021211	398	5.67	45.66
29	AcMYB29	Achn089571	250	5.32	28.36
30	AcMYB30	Achn121951	290	6.25	31.95
31	AcMYB31	Achn109411	360	9.5	41.2
32	AcMYB32	Achn204961	315	6.44	35.54
33	AcMYB33	Achn370001	573	4.9	62.43
34	AcMYB34	Achn368681	1867	5.14	204.94
35	AcMYB35	Achn384891	407	6.35	44.43
36	AcMYB36	Achn345001	382	5.57	43.42
37	AcMYB37	Achn132371	275	5.22	31.41
38	AcMYB38	Achn182891	267	5.95	29.99
39	AcMYB39	Achn313331	320	6.33	35.73
40	AcMYB40	Achn307261	252	5.96	29.13
41	AcMYB41	Achn111201	985	7.84	110.4
42	AcMYB42	Achn092691	258	5.52	29.29
43	AcMYB43	Achn001581	250	5.93	28.31
44	AcMYB44	Achn281601	177	6.57	19.86
45	AcMYB45	Achn081171	267	5.83	30.55
46	AcMYB46	Achn229071	399	6.25	43.28

81	AcMYB47	Achn267491	340	6.77	38.23
48	AcMYB48	Achn108411	299	8.86	34.6
49	AcMYB49	Achn031311	318	5.54	36.03
50	AcMYB50	Achn109311	428	6.96	47.9
51	AcMYB51	Achn111951	249	6.02	28.66
52	AcMYB52	Achn035581	510	6.31	57.77
53	AcMYB53	Achn337181	472	8.47	51.48
54	AcMYB54	Achn012091	479	9.34	54.55
55	AcMYB55	Achn215991	418	7.79	46.64
56	AcMYB56	Achn256021	309	9.6	34.95
57	AcMYB57	Achn324811	274	5.59	31.46
58	AcMYB58	Achn077281	429	5.31	48.74
59	AcMYB59	Achn172271	290	5.97	33.88
60	AcMYB60	Achn307151	381	5.79	41.46
61	AcMYB61	Achn098041	443	8.17	49.58
62	AcMYB62	Achn020551	297	7.31	33.38
63	AcMYB63	Achn030851	248	5.15	28.37
64	AcMYB64	Achn163941	516	8.51	56.52
65	AcMYB65	Achn223911	562	5.22	61.11
66	AcMYB66	Achn116791	215	9.45	24.85
67	AcMYB67	Achn222811	330	6.06	37.31
68	AcMYB68	Achn102731	320	5.11	36.89
69	AcMYB69	Achn284401	290	9.51	33.44
70	AcMYB70	Gene.2893	217	8.83	24.15
71	AcMYB71	Achn289001	255	6.85	29.85
72	AcMYB72	Achn051601	389	4.99	44.17
73	AcMYB73	Gene.4278	279	8.58	31.03
74	AcMYB74	Achn173251	322	5.4	36.28
75	AcMYB75	Gene.6x1	221	9.26	25.45
76	AcMYB76	Achn361141	1944	5.28	213.07
77	AcMYB77	Gene.4723	210	7.91	23.1
78	AcMYB78	Achn228371	276	6.35	31.87
79	AcMYB79	Achn195411	295	6.07	33.87
80	AcMYB80	Achn382461	235	8.93	27.14
81	AcMYB81	Achn375641	433	8.66	48.33
82	AcMYB82	Achn195891	197	8.82	22.9
83	AcMYB83	Achn169441	242	6.67	27.39
84	AcMYB84	Achn202121	390	5.44	44.35
85	AcMYB85	Achn317571	254	5.22	28.7
86	AcMYB86	Achn005711	275	5.04	31.33
87	AcMYB87	Achn380841	453	6.31	51.05
88	AcMYB88	Achn007681	458	6.39	49.93
89	AcMYB89	Achn380251	592	8.91	66.07
90	AcMYB90	Achn199471	272	8.42	30.76
91	AcMYB91	Achn040631	358	9.57	41.18
92	AcMYB92	Achn366791	296	8.95	33.26
93	AcMYB93	Achn144741	329	8.58	37.08

Supplementary Table S2. Conserved amino acid motifs of R2R3 AcMYBs from interProScan

**Motif No.**	**Width**	**Conserved amino acid sequences**	**Annotation**
1	41	RCGKSCRLRWINYLRPDIKRGNFTEEEEELIIQLHAIYGNR	Myb domain
2	22	WAAIAAHLPGRTDNEIKNYWNT	Myb domain
3	15	GPWTPEEDQKLINYV	Myb domain
4	15	HGHGCWRAVPKLAGL	/
5	21	HIKKKLIKMGIDPVTHKPLTD	/
6	15	YGPRNWNLIAQKIPG	Myb domain
7	11	MGRQPCCDKVG	/
8	50	QCGHGCCEAESGGVPRRSLLGPEFVEYEEPPPFPSHEFAAI ATDINNIAW	/
9	29	WKKIAECFPDRTDVQCLHRWQKVLNPELV	Myb domain
10	34	FTPYCVWHTPFRCHSPEAVLKSAAMSFMCTPSIL	/

Note: These conserved amino acid sequences were identified by using the MEME program (http://meme.ncbr.net/meme/cgi-bin/meme.cgi) and subsequently annotated with interProScan (http://www.ebi.ac.uk/Tools/pfa/iprscan/).

Supplementary Table S3. Expression levels of R2R3-*AcMYB* genes during kiwifruit development

Gene	S1	S2	S3	S4	S5	S6	S7
AcMYB1	1.894	0.604	0.664	1.316	1.344	1.332	2.041
AcMYB2	0	0	0	0	0	0	0
AcMYB3	2.115	3.274	2.093	2.308	2.363	2.052	2.457
AcMYB4	2.014	0.561	0	0.483	0.654	1.67	2.67
AcMYB5	1.594	2.108	0.854	1.989	1.481	1.785	1.429
AcMYB6	2.679	2.407	1.835	2.295	2.397	2.812	2.038
AcMYB7	4.236	4.09	2.414	3.057	3.858	3.18	2.753
AcMYB8	2.443	2.329	0.816	1.076	2.069	1.962	2.66
AcMYB9	0	0	0	0	0	0	0
AcMYB10	4.474	1.785	2.955	0.813	1.654	0.943	0.754
AcMYB11	2.414	1.783	0	0	0.797	0	0
AcMYB12	0.617	2.603	2.382	0.68	1.508	0	0
AcMYB13	0.812	0	0	0	0	0	0
AcMYB14	0.657	0	0	0	0	0	0
AcMYB15	1.844	2.895	4.695	3.524	2.909	0.6	0.822
AcMYB16	2.994	0.98	0	0.628	0	0.617	0
AcMYB17	0	0	0.464	0	0.491	1.666	1.593
AcMYB18	0	0	0	0	0	0	0
AcMYB19	5.139	6.433	6.473	5.992	5.878	6.446	5.26
AcMYB20	3.815	0.29	2.456	0.972	0.894	0	1.242
AcMYB21	6.542	0	0.662	0.499	0	0	1.081
AcMYB22	1.842	1.838	2.141	1.66	1.536	1.673	1.697
AcMYB23	0	0	0	0	0	0	0
AcMYB24	5.405	0	0	0.504	0	0	0
AcMYB25	2.605	2.485	2.494	2.955	2.409	1.552	2.057
AcMYB26	0.288	2.097	0	0.328	0.778	1.22	0.419
AcMYB27	1.592	1.64	1.988	1.952	0.955	1.838	0.932
AcMYB28	0.223	0	0	0	0	0	0
AcMYB29	2.274	0.483	0	0	0	0	0
AcMYB30	1.835	0	0	0	0	0.624	0
AcMYB31	5.714	4.584	3.851	3.479	4.616	3.882	3.806
AcMYB32	3.09	3.692	3.385	3.591	3.642	4.242	4.365
AcMYB33	0.344	0	0.648	0.197	0.678	0.977	0.255
AcMYB34	3.116	2.783	2.984	3.024	2.654	2.524	3.129
AcMYB35	0.313	0.207	0	0	0	0.397	0
AcMYB36	0.234	0	0	0	0	0	0
AcMYB37	1.646	0	0	0	0	0	0
AcMYB38	0.411	0	0	0	0	0	0
AcMYB39	0	0	0	0	0	0	0
AcMYB40	0	0	0	0	0	0	0
AcMYB41	2.764	0.749	0.469	0.573	0	1.321	0.62
AcMYB42	0	0	0	0	0	0	0
AcMYB43	0	0	0	0	0	0	0
AcMYB44	0	0	0	0	0	0	0
AcMYB45	0	0	0	0	0	0	0
AcMYB46	0.859	0	0	0	0	0	0

AcMYB47	2.85	0	0	0	0	0	0
AcMYB48	0	0.486	0	0	0	0	0
AcMYB49	0	0	0	0	0	0	0
AcMYB50	1.816	2.396	2.649	1.76	1.435	1.736	1.669
AcMYB51	0	0	0	0	0	0	0
AcMYB52	1.596	2.138	1.617	1.851	1.306	2.334	1.685
AcMYB53	1.831	1.22	1.811	1.571	1.116	1.15	1.273
AcMYB54	3.798	1.6	2.073	3.577	5.873	8.995	8.597
AcMYB55	1.216	0.875	0.829	1.117	0.276	0.358	0
AcMYB56	2.378	0	0	0	0	0	0
AcMYB57	3.943	3.687	3.501	1.995	3.434	3.125	1.009
AcMYB58	7.548	9.495	9.452	8.824	9.391	9.235	8.979
AcMYB59	0.653	1.322	1.643	0.967	0.933	1.098	0
AcMYB60	3.932	2.435	2.824	3.064	3.179	2.374	2.555
AcMYB61	3.841	5.656	6.699	7.207	7.245	7.828	7.491
AcMYB62	3.227	0	0	0.391	0	0	0
AcMYB63	0	0	0	0	0	0	0
AcMYB64	0.767	0.276	1.144	1.321	0.961	1.124	0.823
AcMYB65	1.945	0.748	1.099	0.913	1.152	0.594	0.48
AcMYB66	0.873	0	0	0	0	0	0
AcMYB67	0	0	0	0	0	0	0
AcMYB68	4.27	0.439	1.62	0	0	0	0
AcMYB69	0	0	0	0	0	0	0
AcMYB70	4.763	2.831	1.876	0.363	0	0	0
AcMYB71	0	0	0	0	0	0	0
AcMYB72	5.628	6.462	6.663	6.331	6.244	6.159	6.223
AcMYB73	5.443	6.104	6.225	5.896	6.222	5.823	6.062
AcMYB74	0	0	0	0	0	0	0
AcMYB75	3.801	7.559	6.862	6.475	7.649	6.448	7.282
AcMYB76	5.153	4.932	5.968	4.809	4.656	4.288	4.321
AcMYB77	4.358	1.949	3.473	1.443	1.31	0	0
AcMYB78	1.581	1.144	0.948	1.572	0.967	2.177	2.051
AcMYB79	1.085	1.056	0	1.139	1.196	1.379	0
AcMYB80	0	1.308	2.747	1.162	1.122	0	0
AcMYB81	0	0	0	0	0	0	0
AcMYB82	1.561	0	0	0	0.953	0	0
AcMYB83	0	0	0	0	0	0	0
AcMYB84	0	0	0	0	0	0	0
AcMYB85	0	0	0	0	0	0	0
AcMYB86	1.274	0.806	0.51	0	0	0	0
AcMYB87	0	0	0	0	0	0	0
AcMYB88	2.2	0.872	0.553	1.689	0.802	0.421	0.821
AcMYB89	0.964	1.282	1.58	1.948	1.226	1.477	1.047
AcMYB90	3.003	1.419	1.112	0.89	0	0	0
AcMYB91	5.469	5.632	3.674	4.781	5.597	6.001	5.613
AcMYB92	2.376	1.804	1.188	1.519	1.872	1.731	1.405
AcMYB93	0	0	0	0	0	0	0

Note: The expression of R2R3 MYB genes was normalized and calculated as FPKM

Supplementary Table S4: The genes for qRT-PCRQRT-PCR in *Arabidopsis*

Gene	Locus	Forward primer	Reverse primer
Actin 2	At3g18780	CCGGTATTGTGCTGGATTCTGG	CCAGCAAGGTCAGAGCGGAGG
GAPDH	At3g22650	CTTCTGCAATGGGAAGCAGTGG	GGAGAGTCCTTGCGACCATGC
CHS	At5g13930	GCGCATGTGCGACAAGTCGAC	CTTGATGGCCTTCACTGCCG
CHI	At3g55120	CCGGTGTCCGAGGCCTTGAT	CTTCTCAAACGCACCGGTGAC
F3H	At3g51240	ACCTCCAGGGAGAGGCTGTGC	GCCTCTCACTATACTCCTCCGG
F3'H	At5g07990	CGACAGGAAGAGGTTGGAACGC	GCCGAACAATCGCCGTCCGAT
DFR	At5g42800	CACTCGGAACGAGGCGCATTAC	CAACACCTTCAAACGTTGAAGGTAC
ANS	At4g22880	CATAGAAGCAACGAGTGAGTACGC	CCACACCGAGTGCTAGCTCAG
F3GT	At5g17050	CGGTGTCAAAGAAGTAGGTGAGC	CAGCAGTGGCACGAGGCAAAG
