# Supplementary material for: Kiwifruit R2R3-MYB transcription factors and contribution of the novel AcMYB75 to red kiwifruit anthocyanin biosynthesis
Source: Sci Rep. 2017 Dec 4;7:16861. doi: 10.1038/s41598-017-16905-1 (PMC5715094; doi:10.1038/s41598-017-16905-1)
Supplement: Supplementary file 2 — Supplementary Figures [file 41598_2017_16905_MOESM2_ESM.pdf]

Supplementary materials

Kiwifruit R2R3-MYB transcription factors and contribution of the novel *AcMYB75* to red kiwifruit anthocyanin biosynthesis

Wenbin Li[1 †], Zehong Ding[1 †], Mengbin Ruan[1], Xiaolin Yu[1], Ming Peng[1*], Yifei Liu[2*]

Supplementary Fig. S1

This analysis was performed in Integrative Genomics Viewer (IGV). For each of the five AcMYBs (AcMYB20, AcMYB70, AcMYB73, AcMYB77, and AcMYB75), lots of pair-end reads were mapped on a genome region without gene annotation, strongly supporting that they are novel AcMYBs and not previously identified.

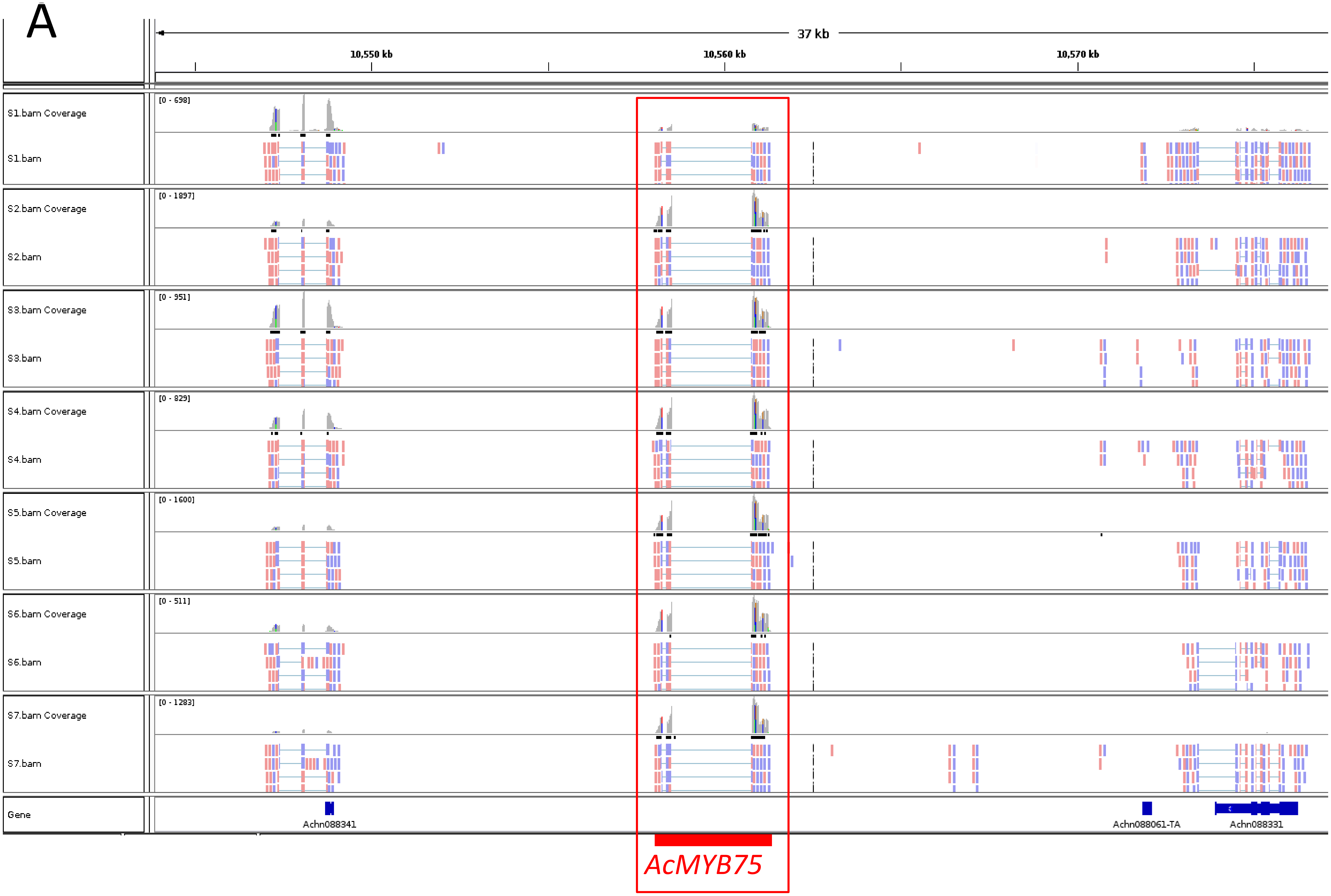

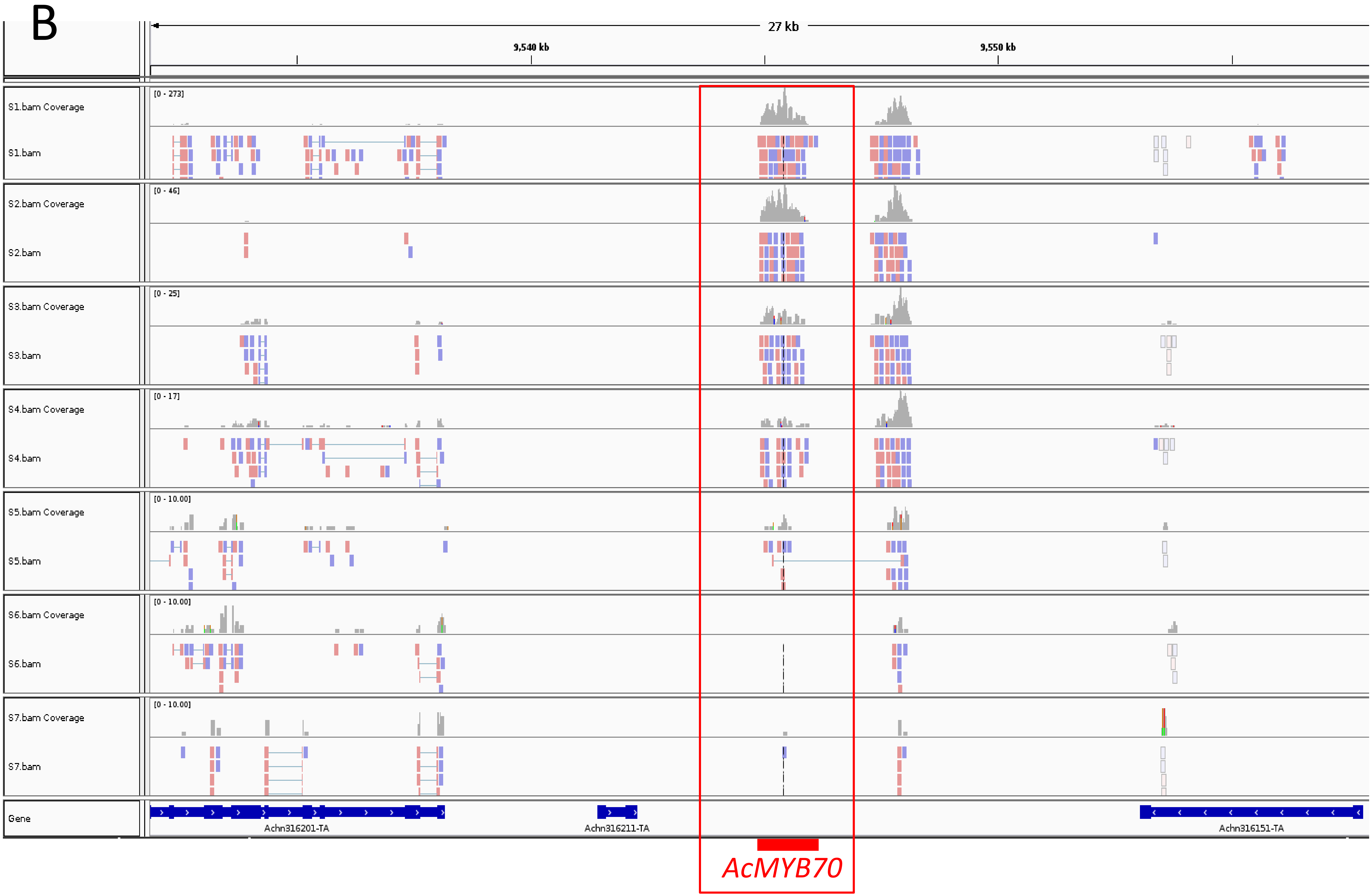

B
27 kb
9,540 kb
9,550 kb
S1.bam Coverage
[0 - 273]
S1.bam
S2.bam Coverage
[0 - 46]
S2.bam
S3.bam Coverage
[0 - 25]
S3.bam
S4.bam Coverage
[0 - 17]
S4.bam
S5.bam Coverage
[0 - 10.00]
S5.bam
S6.bam Coverage
[0 - 10.00]
S6.bam
S7.bam Coverage
[0 - 10.00]
S7.bam
Gene
Achn316201-TA
Achn316211-TA
Achn316151-TA
AcMYB70

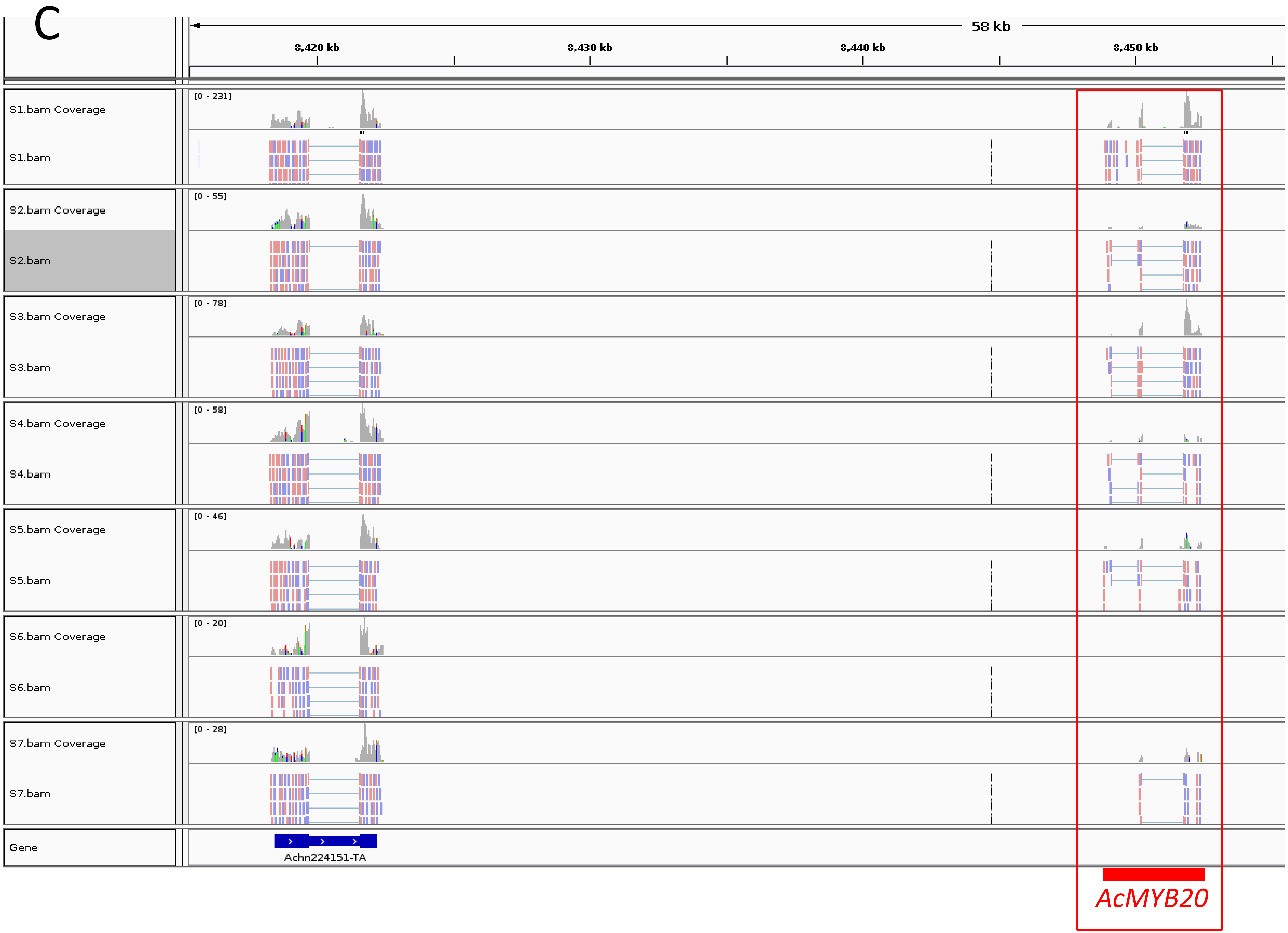
C
58 kb
8,420 kb
8,430 kb
8,440 kb
8,450 kb
S1.bam Coverage
[0 - 231]
S1.bam
S2.bam Coverage
[0 - 55]
S2.bam
S3.bam Coverage
[0 - 78]
S3.bam
S4.bam Coverage
[0 - 58]
S4.bam
S5.bam Coverage
[0 - 46]
S5.bam
S6.bam Coverage
[0 - 20]
S6.bam
S7.bam Coverage
[0 - 28]
S7.bam
Gene
Achn224151-TA
AcMYB20

D

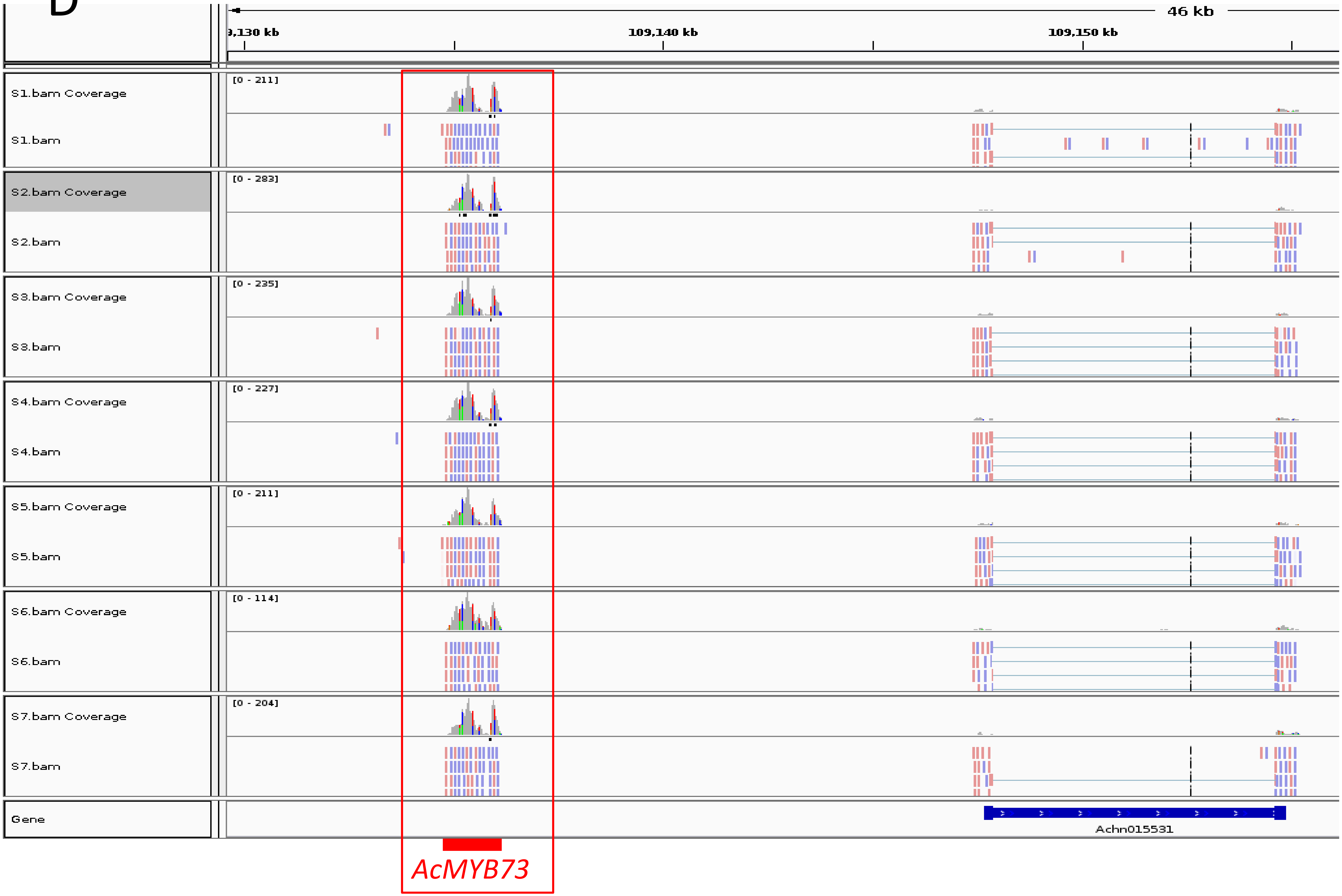
46 kb
9,130 kb
109,140 kb
109,150 kb
S1.bam Coverage
S1.bam
[0 - 211]
S2.bam Coverage
S2.bam
[0 - 283]
S3.bam Coverage
S3.bam
[0 - 235]
S4.bam Coverage
S4.bam
[0 - 227]
S5.bam Coverage
S5.bam
[0 - 211]
S6.bam Coverage
S6.bam
[0 - 114]
S7.bam Coverage
S7.bam
[0 - 204]
Gene
Achn015531
AcMYB73

E

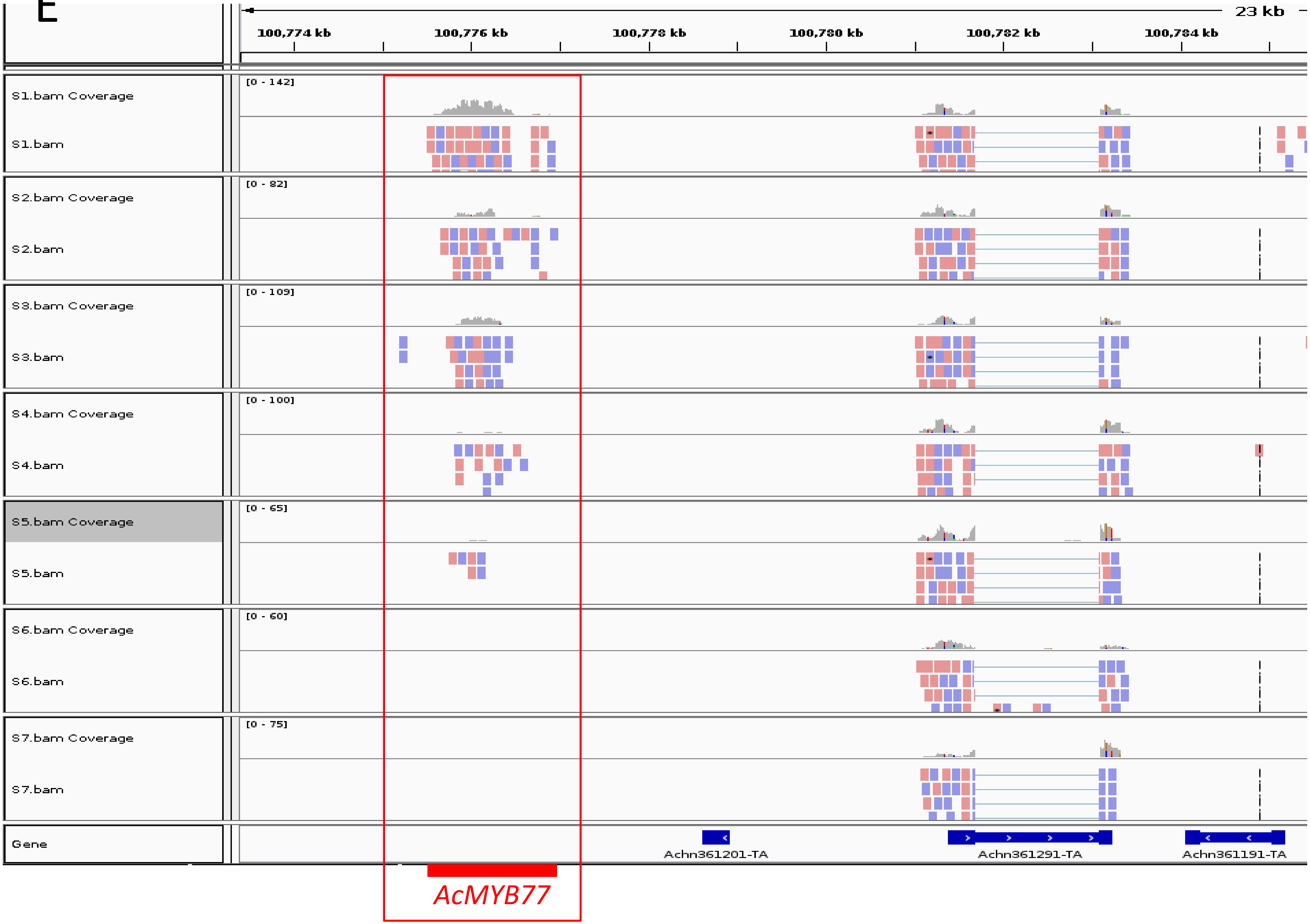
23 kb
100,774 kb
100,776 kb
100,778 kb
100,780 kb
100,782 kb
100,784 kb
S1.bam Coverage
[0 - 142]
S1.bam
S2.bam Coverage
[0 - 82]
S2.bam
S3.bam Coverage
[0 - 109]
S3.bam
S4.bam Coverage
[0 - 100]
S4.bam
S5.bam Coverage
[0 - 65]
S5.bam
S6.bam Coverage
[0 - 60]
S6.bam
S7.bam Coverage
[0 - 75]
S7.bam
Gene
Achn361201-TA
Achn361291-TA
Achn361191-TA
AcMYB77

Supplementary Fig. S2

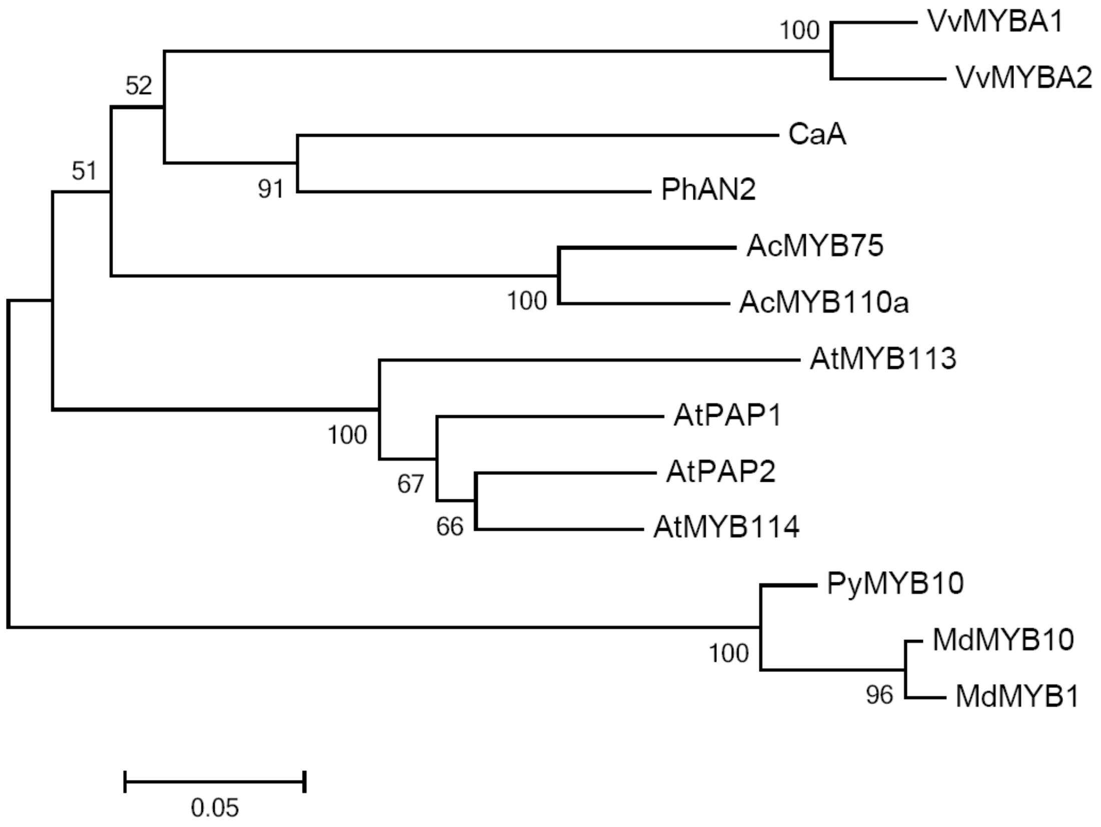

The alignment of amino acids from Fig. 4 was conducted with Clusta W. A linearized neighbor-joining tree was produced with MEGA software version 5.05. Bootstrap values for nodes are shown as numbers on branches. Evolutionary distances (Poisson-correction method) are shown as number of substitutions per amino acid site.

Supplementary Fig. S3

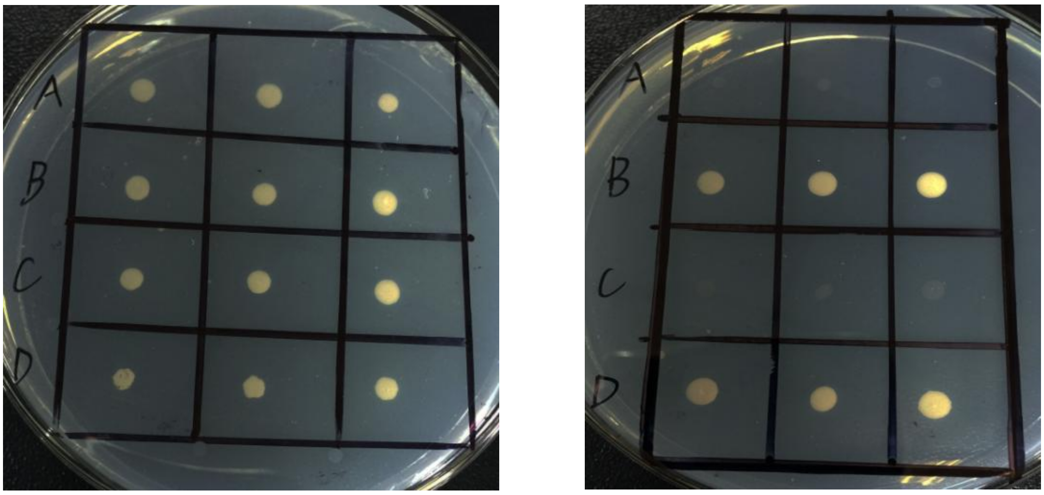

The yeast two hybrid screening between AcMYB75 and AtTT8 (At4g09820)
Left: SD/-Leu/-Trp; Right: SD/-Leu/-Trp/-His/-Ade. Three replicates
(A): BD-lam/AD-T;
(B): BD53/AD-T;
(C): AtTT8-1-BD/AcMYB75-AD (AtTT8-1 is from 58-603 bp of AtTT8 for bHLH-MYC_N superfamily);
(D): AtTT8-N-BD/AcMYB75-AD (AtTT8-N is from 1-603 bp of AtTT8)

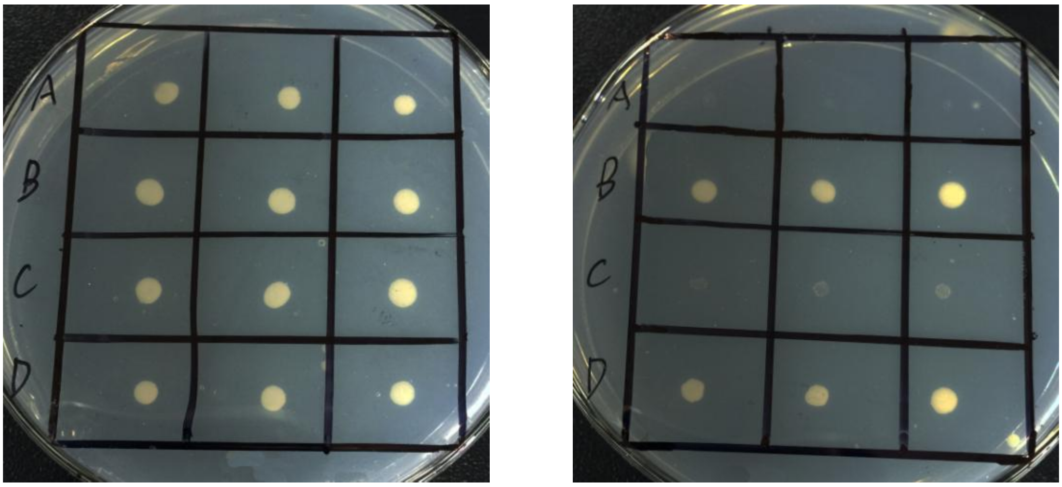

The yeast two hybrid screening between AcMYB75 and AtGL3 (At5g41315)
Left: SD/-Leu/-Trp; Right: SD/-Leu/-Trp/-His/-Ade. Three replicates
(A): BD-lam/AD-T;
(B): BD53/AD-T;
(C): AtGL3-1-BD/AcMYB75-AD (AtGL3-1 is from 55-633 bp of AtGL3 for bHLH-MYC_N superfamily);
(D): AtGL3-N-BD/AcMYB75-AD (AtGL3-N from 1-633 bp of AtGL3).
